# LY6K is a novel molecular target in bladder cancer on basis of integrate genome-wide profiling

**DOI:** 10.1038/sj.bjc.6605990

**Published:** 2010-11-09

**Authors:** R Matsuda, H Enokida, T Chiyomaru, N Kikkawa, T Sugimoto, K Kawakami, S Tatarano, H Yoshino, K Toki, Y Uchida, K Kawahara, K Nishiyama, N Seki, M Nakagawa

**Affiliations:** 1 Department of Urology, Graduate School of Medical and Dental Sciences, Kagoshima University, 8-35-1 Sakuragaoka, Kagoshima 890-8520, Japan; 2 Department of Functional Genomics, Graduate School of Medicine, Chiba University, Chiba, Japan; 3 Kawahara Nephro-urology Clinic, Kagoshima, Japan

**Keywords:** LY6K, bladder cancer, array-CGH, 8q24.3

## Abstract

**Background:**

The aim of this study is to find a novel molecular target based on chromosomal alteration and array-based gene expression analyses in bladder cancer (BC). We investigated a cancer testis antigen, *LY6K*, which is located on chromosome 8q24.3.

**Methods:**

Five BC cell lines were subjected to high-resolution array-comparative genomic hybridisation with 244 000 probes. The expression levels of *LY6K* mRNA were evaluated in BC cell lines and clinical BC specimens by real-time reverse transcription–PCR. The cell lines were subjected to fluorescence *in situ* hybridisation of *LY6K*. Cell viability was evaluated by cell growth, wound healing, and matrigel invasion assays.

**Results:**

Typical gained loci (*P*<0.0001) at 6p21.33-p21.32, 8q24.3, 9q34.13, 11q13.1-q14.1, 12q13.12-q13.13, 16p13.3, and 20q11.21-q13.33 were observed in all of the cell lines. We focused on 8q24.3 locus where *LY6K* gene harbours, and it was the top upregulated one in the gene profile from the BC cell line. *LY6K* mRNA expression was significantly higher in 91 BCs than in 37 normal bladder epitheliums (*P*<0.0001). Fluorescence *in situ* hybridisation validated that the high *LY6K* mRNA expression was due to gene amplification in the region where the gene harbours. Cell viability assays demonstrated that significant inhibitions of cell growth, migration, and invasion occured in LY6K knock down BC cell lines; converse phenomena were observed in a stable *LY6K* transfectant; and LY6K knockdown of the transfectant retrieved the original phenotype from the *LY6K* transfectant.

**Conclusion:**

Upregulation of the oncogenic *LY6K* gene located on the gained locus at 8q24.3 may contribute BC development.

Bladder cancer (BC) is among the five most common malignancies worldwide, and it is the second most common tumour of the genitourinary tract and the second most common cause of death in patients with genitourinary tract malignancies (Jemal *et al*, 2009). In Japan, the age-standardised mortality rate of BC patients has increased slightly since 1993 (Qiu *et al*, 2009). The 5-year survival rate of patients with non-muscle-invasive BC is close to 90%, whereas that with muscle-invasive BC is ∼60%. In spite of various therapeutic treatments, more than 90% of patients with metastasis die within the first 5 years (Luke *et al*, 2009). Therefore, new diagnostic methods and new treatments for BC are urgently needed.

Comparative genomic hybridisation (CGH) has facilitated chromosomal characterisation of solid tumours, as it can provide detailed information on gain and loss of tumour DNA across the entire genome (Inazawa *et al*, 2004; Ishkanian *et al*, 2004). Conventional CGH is widely used to analyse many types of tumours, including BC (Hovey *et al*, 1998; Zhao *et al*, 1999; Simon *et al*, 2000; von Knobloch *et al*, 2000; Mahdy *et al*, 2001). Recently, microarray-based CGH has been used by several groups to study copy number instability and aberration type in BC specimens (Stoehr *et al*, 2004; Blaveri *et al*, 2005; Shinoda *et al*, 2007; Yamamoto *et al*, 2007; Hurst *et al*, 2008). According to the previous BC studies, frequent copy number gains have been observed at 1q, 3p, 3q, 5p, 6p, 8q, 10p, 11q, 12q, 17q, 19q, and 20q, whereas copy number losses have been observed at 2q, 4q, 5q, 8p, 9p, 9q, 10q, 11p, 11q, 13q, and 18q. These studies also demonstrated that the aberration patterns characterise invasive or non-invasive BC (Hovey *et al*, 1998; Zhao *et al*, 1999; Simon *et al*, 2000; Mahdy *et al*, 2001; Shinoda *et al*, 2007; Blaveri *et al*, 2009), and that they could be progression markers for BC (von Knobloch *et al*, 2000; Stoehr *et al*, 2004; Yamamoto *et al*, 2007). Their findings suggest that promising candidates for tumour-related genes might be located where the aberrations occur. However, identification of tumour-related genes is difficult because many genes are involved in the larger chromosomal areas.

Gene expression profiling by oligonucleotide microarray analysis is an excellent tool for screening candidate genes that have a tumour suppressive or oncogenic function in BC (Dyrskjot *et al*, 2003; Kawakami *et al*, 2006). We previously found by microarray analysis that *SKP2* and *CKS1* contribute to progression and prognosis in BC (Kawakami *et al*, 2007). However, the hundreds of candidate genes identified by microarray analysis can make it difficult for investigators to decide which genes to study. Expression analysis of genes located in regions of gains or losses has shown that the gene expression level changes along with the gene copy number (Inazawa *et al*, 2004; Ishkanian *et al*, 2004). For example, comparisons of array-CGH and transcriptome data have shown that 40–60% of the genes in highly amplified regions are actually overexpressed (Pollack *et al*, 2002; Heidenblad *et al*, 2005). Therefore, genome amplifications and homozygous deletions could be landmarks in cancer cell genomes for identifying oncogenes and tumour suppressor genes, respectively. Our group previously integrated array-CGH data analysis with gene expression profiling to identify candidate genes with oncogenic function in squamous cell carcinoma (Sugimoto *et al*, 2009). We have now used high-resolution array-CGH, performed using about 244 000 probes with a length of 60-mer. This proved to be a powerful profiling tool because it detected more deletions and smaller regions of gains.

*LY6K*, which is located at 8q24.3, belongs to the *LY6* family. It shows high homology to the low-molecular-weight glycosyl-phosphatidyl-inositol-anchored molecule, which is either a cell surface receptor or a secreted granule involved in the cell signaling pathway (de Nooij-van Dalen *et al*, 2003). It is also a cancer/testis antigen, that is, a protein highly expressed in cancer cells but not in normal cells, except for testis (Scanlan *et al*, 2004; Ishikawa *et al*, 2007; Kono *et al*, 2009). Several groups have shown elevated expression of *LY6K* mRNA in human head and neck squamous cell carcinomas and in lung, oesophageal, and breast cancers (de Nooij-van Dalen *et al*, 2003; Lee *et al*, 2006; Ishikawa *et al*, 2007). However, little is known about the functional role of LY6K in BC development. We evaluated *LY6K* mRNA expression levels in clinical BC samples and established a stable *LY6K* transfectant for functional analysis of the gene.

## Materials and methods

### Cell lines and culture

We used five human BC cell lines: BOY was established in our laboratory from an Asian male patient aged 66 diagnosed with stage III BC with lung metastasis (Takemoto *et al*, 1997); T24, UMUC, and J82 were obtained from the American Type Culture Collection; and KK47 was established at Kanazawa University (Imao *et al*, 1999) and kindly provided. These cell lines were maintained in a minimum essential medium (Sigma-Aldrich, St Louis, MO, USA) supplemented with 10% fetal bovine serum (FBS; Equitech-Bio Inc., Kerrville, TX, USA) in a humidified atmosphere of 5% CO_2_ and 95% air at 37°C.

### Array-CGH analysis of BC cell lines

Chromosomal DNA was isolated from the five cell lines using a FlexiGene (Qiagen, Hilden, Germany) in accordance with the manufacturer's protocol. The purity and molecular weight of the DNA were estimated using agarose gels. The Human Genome CGH Microarray 244K (Agilent Technologies, Palo Alto, CA, USA), which contains over 244 000 60-mer oligonucleotide probes, spanning coding and non-coding genomic sequences with median spacing of 7.4 and 16.5 kb, respectively, was used for copy number measurement. Human genomic DNA (Novagen, Madison, WI, USA) was used as a reference. Labelling, hybridisation, and scanning were performed in accordance with the manufacturer's protocol. Common aberrant loci were detected using CGH Analytics Software (Agilent Technologies) with the ADM2 algorithm (∣log2 ratio∣>0.3, *P*-value <0.05, overlap <0.5; de Smith *et al*, 2007). The array-CGH data from this study have been submitted to the NCBI Gene Expression Omnibus (GEO; http://www.ncbi.nlm.nih.gov/geo) under accession no. GSE19714.

### Oligonucleotide microarray analysis of BC cell lines

Total RNA was extracted using TRIzol reagent (Invitrogen, Carlsbad, CA, USA) in accordance with the manufacturer's protocol. The integrity of the RNA was checked by a RNA 6000 Nano Assay kit and a 2100 Bioanalyzer (Agilent Technologies). The Whole Human Genome Microarray 44K (no. G4112F, Agilent Technologies), which contains over 41 000 60-mer oligonucleotide probes (GEO platform ID: GPL4133), was used for expression profiling. Hybridisation and washing were performed in accordance with the instructions of the manufacturer. Because no normal bladder epithelium cell line is commercially available, we used human bladder total RNA (Clontech, Mountain View, CA, USA) as a reference for microarray analysis. The arrays were scanned using a Packard GSI Lumonics ScanArray 4000 (Perkin Elmer, Boston, MA, USA). The data obtained were analysed by means of DNASIS array software (Hitachi Software Engineering, Yokohama, Japan), which converted the signal intensity for each spot into text format. The data from each microarray study were normalised using glucuronidase-*β* (*GUSB*) and expressed in absolute numbers. The oligonucleotide array data are available for reference (NCBI GEO; http://www.ncbi.nlm.nih.gov/geo; under accession no. GSE19716).

### Clinical samples

In all, 91 tissue samples were obtained from BC patients who had undergone surgical resection at our institution between 2003 and 2007. Also used were 37 pathologically proven normal bladder epithelium (NBE) samples derived from organ-confined prostate cancer patients who underwent prostatectomy. The background and clinicopathological characteristics of the patients are summarised in Table 1. These 128 samples were used for quantitative real-time reverse transcription PCR (RT–PCR). They were staged in accordance with the American Joint Committee on Cancer Union Internationale Contre le Cancer tumour–node–metastasis classification and histologically graded (Sobin and Wittekind, 2002). Our study was approved by the Bioethics Committee of Kagoshima University; written previous informed consent and approval were given by the patients.

### Sample preparation and total RNA extraction

Freshly harvested tissues, immediately frozen in liquid nitrogen and stored at −80°C, were dissolved in TRIzol reagent (Invitrogen) for total RNA extraction following the protocol of the manufacturer. Total RNA from peripheral blood lymphocytes (PBLs) of BC patients were extracted by RNeasy Mini Kit (no. 74106, Qiagen). RNA density was measured with an Ultrospec 3100 Pro instrument (Amersham Biosciences, Piscataway, NJ, USA), and RNA quality was checked with the 2100 Bioanalyzer (Agilent Technologies).

### Complementary DNA (cDNA) preparation and quantitative real-time RT–PCR

First strand cDNA with 1 *μ*g total RNA was synthesised using oligo-deoxythymidine primers of the RT system (Promega, Tokyo, Japan). Gene-specific PCR products were assayed continuously using a 7900 real-time PCR system (Applied Biosystems, Foster City, CA, USA) in accordance with manufacturer's protocol. The initial PCR step was a 10-min hold at 95°C. The cycles (*n*=40) consisted of a 15-s denaturation step at 95°C, followed by 1 min annealing/extension at 63°C. All reactions were performed in triplicate. For quantitative analysis, *GUSB* served as an internal control. The TaqMan probes and primers used for *LY6K* and *GUSB* were assay-on-demand gene expression products (Applied Biosystems). The gene expression relative to normal bladder RNA (human bladder total RNA, Clontech) was calculated using the comparative *C*t method.

### Fluorescence *in situ* hybridisation (FISH) analysis

A FISH analysis of *LY6K* amplification was applied to five BC cell lines with 5-*μ*M thick paraffin-embedded tissues. For FISH probes, the *LY6K* locus-containing bacterial artificial chromosome probes (clone ID; RP11-119A16, RP11-163E23) and the chromosome 8 centromere probe (KBI-20008G, Kreatech Diagnosis, Amsterdam, The Netherlands) were labelled using a nick translation kit (Vysis 32-801300, Abbott Molecular Inc., Des Plaines, IL, USA) with orange-dUTP (Abbott molecular inc.) and PlatinumBright 495 (Kreatech Diagnosis), respectively. Denaturation, hybridisation, and post-hybridisation washing were carried out in accordance with Poseidon protocol (Kreatech Diagnosis). The specimens were counterstained with 4,6-dianidino-2-phenylindone (DAPI) and examined using a PM-2000 imaging system (HistoRx, New Haven, CT, USA) equipped with a triple filter set (Chroma, Bellows Falls, VT, USA): no. 41007 for orange-dUTP, no. 41017 for Platinum*Bright* 495, and no. 31000v2 for DAPI.

### Immunoblotting

Total protein lysate was prepared with triple detergent lysis buffer composed of 50 mM tris-HCl (pH 8.0), 150 mM NaCl, 0.02% NaN3, 0.1% sodium dodecyl sulphate, 1% NP-40, and 0.5% sodium deoxycholate in the presence of a protease inhibitor cocktail (Sigma-Aldrich) and 100 mM phenylmethylsulfonyl fluoride. The protein lysate (50 *μ*g per lane) was separated by NuPAGE electrophoresis on 4–12% bis-tris gel (Invitrogen) and transferred to a polyvinylidene difluoride membrane. Immunoblotting was carried out with diluted (1:500) polyclonal LY6K antibody (no. IMG_4183, Imgenex, San Diego, CA, USA). After being washed, the membrane was incubated with goat anti-rabbit IgG horseradish peroxidase conjugate (Bio-Rad, Hercules, CA, USA). Specific complexes were visualised with an Amersham ECL Western blotting detection system (GE Healthcare, Little Chalfont, UK).

### Small interfering RNA (siRNA) transfection

We obtained siRNA oligonucleotide target to *LY6K* (small interfering (si)-*LY6K*) from the ON-TARGETplus SMART pool (L-013771-01-0005; Dharmacon, IL, USA) and non-target as control (si-control) from the non-targeting pool (D-001810-10-05; Dharmacon). The T24/*LY6K* transfectant, and two BC cell lines (BOY and KK47) were transfected with 10 nM siRNA using Lipofectamine RNAiMAX (Invitrogen) reagent following the manufacturer's protocol to evaluate the knockdown effect of siRNA by quantitative real-time RT–PCR and western blot analysis.

### Construction of LY6K expression vectors and transfection to T24 cells

The LY6K vector was constructed by inserting full-length *LY6K* cDNA into the BamH I and Hind III restriction sites of the pBApo-CMV Neo vector (Takara Bio, Otsu, Japan). The *LY6K* and non-targeting (control) vectors were transfected into T24 cells by calcium phosphate co-precipitation. We did not subject UMUC, in which the *LY6K* expression level was lower than in T24, because its growth was too slow and it was not suitable for transfection. The T24/*LY6K* transfectants were split and grown in selective medium with 1000 mg l^−1^ of G418. In all, 15 G418-resistant colonies were chosen and expanded in medium containing 200 mg l^−1^ of G418. DNA sequences for all constructs were confirmed by DNA sequencing (Bio Matrix Research Inc., Tokyo, Japan). Finally, we selected the one with the highest *LY6K* mRNA expression among the clones.

### XTT assays

Cells were seeded at a density of 3 × 10^3^ cells per well in 96-well plates and incubated for 48 h. Subsequently, cell viability was determined by the XTT assay following the manufacturer’s protocol (Roche Applied Sciences, Indianapolis, IN, USA). The plates were read using an MPR-A4i microplate reader (Tosho, Tokyo, Japan). All experiments were repeated in triplicate.

### Wound healing assay

Cells (2 × 10^5^) were seeded into six-well plates and cultured in medium containing 10% of FBS to create a confluent monolayer. They were carefully wounded using 200-*μ*l pipette tips and any cell debris was removed with phosphate-buffered saline. Microphotographs were taken, and quantitative analysis of the percentage of wound healing was calculated using the distance across the wound at 0 h and the indicated time for each cell lines. All experiments were repeated in triplicate.

### Invasion assay

*In vitro* invasion assay was done using BD BioCoat Matrigel Invasion Chambers (BD Biosciences, Bedford, MA, USA) in 24-well plates. Cancer cells (5 × 10^4^) were added to the upper chamber, and the lower chamber was filled with conditioned medium. After being incubated for 24 h, cells that migrated through the membrane to the lower surface were stained with Giemsa solution. Four randomly selected × 200 magnification fields were photographed, and the number of invading cells was counted. All experiments were repeated in duplicate.

### Statistical analysis

The relationship between two groups of findings and numerical values obtained by real-time PCR and other assay was analysed with the Mann–Whitney *U*-test. The analysis software was Expert StatView (version 4, SAS Institute Inc., Cary, NC, USA); the box plot style with logarithmic scale was constructed, and the non-adjusted statistical level of significance of *P*-value was <0.05. The 95% confidence interval (CI) was calculated using Microsoft Office Excel (version 2007, Microsoft Corp., Redmond, WA, USA).

## Results

### Genomic profiling of BC cell lines by array-CGH

**Figure 1:**
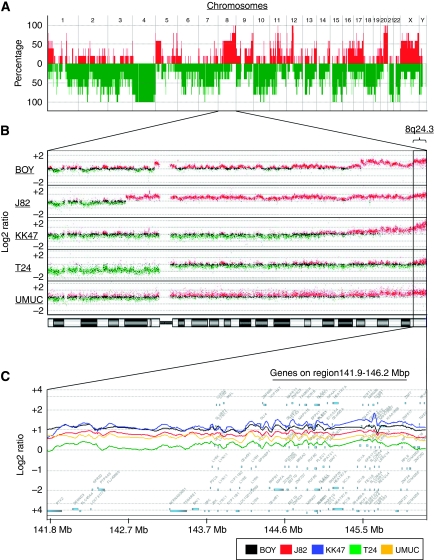
(**A**) Array-CGH profiling of five BC cell lines showing recurrence of chromosomal alterations. Integer value recurrence of copy number alterations in segmented data (*y*-axis) was plotted for each probe aligned along the *x*-axis in chromosome order. Red and green bars denote gain and loss of chromosome material, respectively. The most recurrent regions of DNA copy number gains were on chromosomes 1q, 8q, 11q, and 20q, whereas recurrent regions of copy number loss were on chromosomes 4p, 4q, 10p, 19p, and 21q. (**B**) Magnification of chromosome 8 locus in array-CGH profiling. Red and green denote >0.5 and <0.5 of log2 ratio value. The predominant aberration occurred at 8q24.3. (**C**) Smoothed copy number profile from 143.2 to 145.6 Mb on chromosome 8 (8q24.3) using CGH Analytics Software (Agilent Technologies). A total of 91 genes were located on this region, and *LY6K* was located at 143.8 Mb. Among the genes, 56 were upregulated more than two-fold compared with the reference RNA in the BC cell lines (Table 3).

To produce a comprehensive survey of genomic aberrations in BC, five BC cell lines were analysed using array-CGH for patterns of chromosomal gains and/or losses. Predominantly gained loci were observed at the several loci (Figure 1A, and Supplementary Table SI). Among the gained loci, significant gains (*P*<0.001) at 6p21.33-p21.32, 8q24.3, 9q34.13, 11q13.1-q14.1, 12q13.12-q13.13, 16p13.3, and 20q11.21-q13.33 were observed in all of the cell lines (100% Table 2). On the other hand, lost loci were commonly found at 4p, 4q, 10p, 19p, and 21q in all cell lines (100% Figure 1A, and Supplementary Table SII). We focused on the gain locus at 8q24.3 that was one of the most frequent event in the cell lines (Figure 1B, and Table 2), because it has not been studied in detail for BC. Figure 1C demonstrated that the log ratio of 141.9–146.2 Mb on chromosome 8 (8q24.3) was robustly high in each cell line.

### Genomic copy number and expression profile data at 8q24.3

To identify the genes involved in BC development, we evaluated the expression levels of 91 genes located at 8q24.3 for the five BC cell lines using an oligo-microarray. We identified 56 genes that were upregulated more than two-fold in comparison with the reference RNA (Table 3 upper). The *LY6K* gene was the top upregulated one in the gene profile from the BC cell line with a fold change of 28.62 (relative to normal bladder; Table 3). Hence, we focused on *LY6K* being a promising candidate gene for oncogenic function in BC development. We found that eight LY6 family genes were located on the region between 141.9 and 146.2 Mb on chromosome 8 (8q24.3; Figure 1C). The expression levels of *LY6E*, *GML*, and *LYPD2* were relatively upregulated with an average fold of 6.37, 3.67, and 1.34, whereas those of *LYNX1*, *LY6H*, and *LY6D* were not upregulated in the cell lines (Table 3, bottom).

### Evaluation of *LY6K* mRNA expression and the gene amplification in BC cell lines, and clinical BC samples

**Figure 2:**
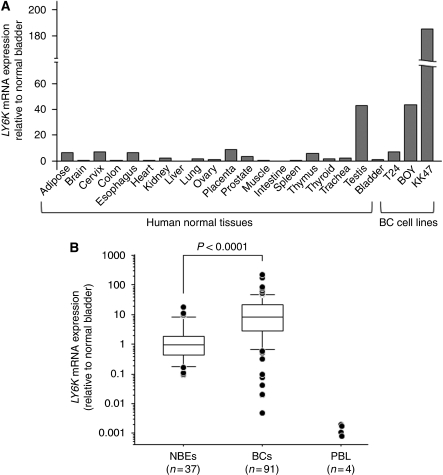
(**A**) *LY6K* mRNA expression levels in a panel of normal tissues and BC cell lines from real-time RT–PCR. Gene expressions relative to normal bladder were calculated using comparative *C*t method. The expression levels were commonly low in human normal tissues except for testis; they were high in two BC cell lines (BOY and KK47). (**B**) *LY6K* mRNA expression was significantly higher in clinical BC samples than in normal bladder epithelium (NBE) samples. Expression levels of *LY6K* mRNA in peripheral blood lymphocytes (PBL) of BC patients (*n*=4) were extremely low. The gene expressions were determined relative to average for NBE samples.

**Figure 3:**
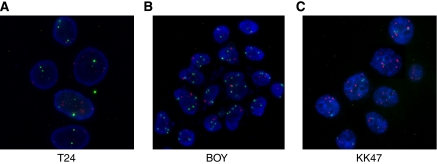
Examples of amplification of *LY6K* gene in BC. Copy number status of gene was determined by fluorescence *in situ* hybridisation (FISH) in (**A**) T24, (**B**) BOY, and (**C**) KK47 BC cell lines. Red signals were from *LY6K* gene probe, and green ones were from centrometric probe (magnification × 200).

Real-time RT–PCR revealed that the expression levels of *LY6K* mRNA were commonly low in human normal tissues except for testis (Figure 2A). In human BC cell lines, *LY6K* mRNA expression was abundant in KK47 (184.9-fold relative to normal bladder), modest in BOY (43.6-fold), and weak in T24 (7.2-fold; Figure 2A). In clinical samples, *LY6K* mRNA expression was significantly higher in the 91 BC samples than in the 37 NBE samples (BC: mean 19.6±3.6, 95% CI 12.4–26.8; NBE 2.4±0.6, 95% CI 1.0–3.7; *P*<0.0001; Figure 2B). When we used a cut-off value of 3.7, which was the upper limit of 95% CI for NBEs, 62 of 91 BCs (68%) were determined to be samples with high *LY6K* mRNA expression. The expression levels of *LY6K* mRNA in (PBLs of BC patients (*n*=4) were extremely low compared with those of BCs and NBEs, indicating that PBL contamination does not increase the level of *LY6K* mRNA expression in BC tissues. We found no relationship between the *LY6K* mRNA expression and clinicopathological parameters (tumour stage, and grade). To investigate whether high *LY6K* mRNA expression depends on gene amplification at the gene location, we performed FISH. The gene amplification of *LY6K* (red signal) in comparison with that of the control centromere probe (green signal) was higher in BOY and KK47 and lower in T24 (Figures 3A–C). These results corresponded with the *LY6K* mRNA expression levels in these cell lines (Figure 2A).

### Effects of LY6K knockdown on cell growth, migration and invasion

**Figure 4:**
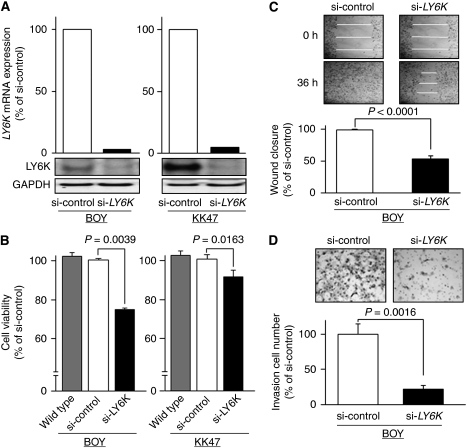
Effects of LY6K knockdown on cell growth. (**A**) *LY6K* mRNA expressions were markedly repressed in si-*LY6K* transfectants (BOY and KK47) in comparison with si-control transfectants. (**B**) Cell viability as determined by XTT assay. Greater growth inhibition was observed in the si-*LY6K* transfectants (BOY and KK47) than in the si-control transfectants. (**C**) Cell migration activity revealed by the wound healing assay. Greater inhibition of cell migration was observed in si-*LY6K* transfectant (BOY) than in the si-control transfectant. (**D**) Cell invasion activity by matrigel invasion assay. The number of cells invading through the membrane was significantly lower in the si-*LY6K* transfectant (BOY) than in the si-control transfectant.

For loss-of-function studies, we used BOY and KK47, in which the *LY6K* mRNA was markedly expressed (Figure 2A). Real-time RT–PCR and immunoblotting demonstrated that *LY6K* mRNA expression was substantially lower after si-*LY6K* transfection to BOY and KK47 (Figure 4A). After 48-h si-*LY6K* transfection to each cell line, XTT assay revealed significant decreases in cell growth in si-*LY6K* transfectants in comparison with si-control transfectants (BOY, 74.1±0.9% and 100±0.6%, respectively, *P*=0.0039; and KK47, 88.2±1.5% and 100±6.5%, respectively, *P*=0.0163; Figure 4B). We found no cytotoxic effect of the transfection reagent in the transfectants compared with the wild types. We subjected BOY to wound healing assay, which can evaluate both cell migration and cell proliferation activity, and matrigel invasion assay. We did not use KK47 because it was not suitable for the experiments because of its focal growth pattern. Wound healing assays demonstrated that the migration into the wound area was slower in the si-*LY6K*-transfected BOY than in the si-control-transfected BOY (54.9±3.6% and 100%, respectively, *P*<0.0001; Figure 4C). Matrigel invasion assay showed that the number of cells invading through the membrane was significantly lower in the si-*LY6K*-transfected BOY than in the si-control-transfected BOY (21.5±5.5% and 100±14.8%, respectively, *P*=0.0016, Figure 4D).

### Effects of LY6K overexpression on cell growth, migration, and invasion

**Figure 5:**
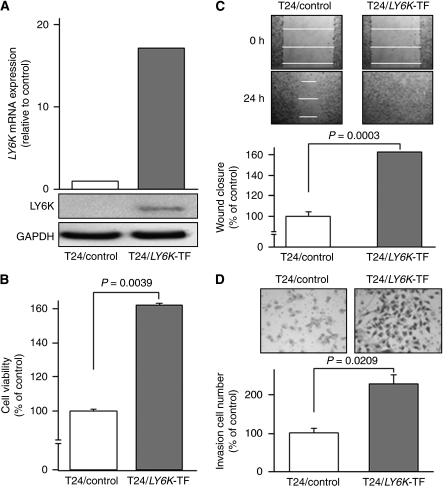
Effects of LY6K overexpression on cell growth, migration, and invasion. (**A**) *LY6K* mRNA and protein expressions in the T24/*LY6K* transfectant (TF) and T24/non-targeting vector transfectant (control). The expression levels of LY6K were markedly higher in the T24/*LY6K*-TF than in the control. (**B**) Cell viability as determined by XTT assay. A significant acceleration of cell growth was observed in the T24/*LY6K*-TF in comparison with the control. (**C**) Cell migration activity by the wound healing assay. Greater acceleration of cell migration was observed in the T24/*LY6K*-TF than in the control. (**D**) Cell invasion activity by matrigel invasion assay. The number of cells invading through the membrane was significantly higher in the T24/*LY6K*-TF than in the control.

Because T24 showed a lower expression of *LY6K* mRNA relative to other cell lines (Figure 2A), we established a stable T24/*LY6K* transfectant for gain of function studies. Real-time RT–PCR showed a high expression level of *LY6K* mRNA in the transfectant, whereas it was weak in the T24/non-targeting vector transfectant (control; Figure 5A). Immunoblotting demonstrated a high expression of 17-kDa LY6K protein in the transfectant, whereas it was undetectable in the control (Figure 5A). Three independent XTT assays consistently demonstrated significant acceleration of cell growth in the transfectant compared with the control (162.1±1.1% and 100±1.3%, respectively, *P*=0.0039; Figure 5B). Wound healing assays demonstrated that the transfectant migrated into the wound area more rapidly than the control (163.4±0% and 100±4.4%, respectively, *P*=0.0003; Figure 5C). Matrigel invasion assay showed that the number of cells invading through the membrane was significantly higher in the transfectant than in the control (228.3±23.4% and 100±11.3%, respectively, *P*=0.0209; Figure 5D).

### Effects of LY6K knockdown in T24/*LY6K* transfectant on cell growth, migration, and invasion

**Figure 6:**
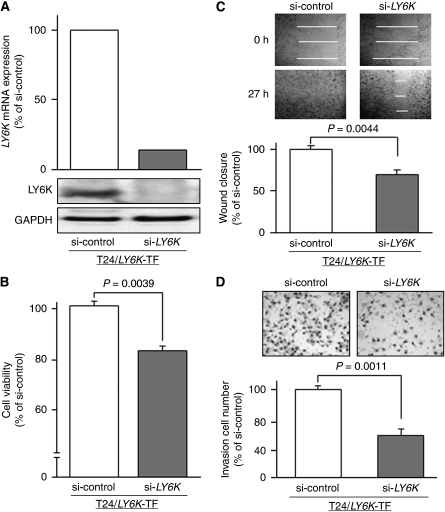
Effects of LY6K knockdown in T24/*LY6K* transfectant on cell growth, migration, and invasion. (**A**) *LY6K* mRNA expressions were markedly lower after si-*LY6K*-transfection of T24/*LY6K* transfectant (TF) than in the si-control transfectant. (**B**) Cell viability as determined by XTT assay. Significant growth inhibition was observed in the si-*LY6K* transfectant. (**C**) Cell migration activity by the wound healing assay. A significant inhibition of cell migration was observed in the si-*LY6K* transfectant. (**D**) Cell invasion activity by the matrigel invasion assay. The number of cells invading through the membrane was significantly decreased in the si-*LY6K* transfectant.

To investigate whether *LY6K* knockdown retrieves the original phenotype of T24 from that of the T24/*LY6K* transfectant, we transfected the transfectant with si-*LY6K* and subjected it to cell viability studies. Real-time RT–PCR and immunoblotting demonstrated that LY6K expression was substantially lower after the si-*LY6K* transfection (Figure 6A). After 48-h si-*LY6K* transfection of the transfectant, XTT assay revealed a significant decrease in cell growth in the si-*LY6K* transfectant compared with the si-control transfectant (84.7±2.0% and 100±1.7%, respectively, *P*=0.0039; Figure 6B). Wound healing assays demonstrated that the migration into the wound area was slower in the si-*LY6K* transfectant than in the si-control transfectant (70.2±3.8% and 100%, respectively, *P*=0.0044; Figure 6C). Matrigel invasion assay showed that the number of cells invading through the membrane was significantly lower in the si-*LY6K* transfectant than in the si-control transfectant (48.2±7.1% and 100±3.6%, respectively, *P*=0.0011; Figure 6D).

## Discussion

In previous studies, chromosomal alteration analysis by CGH and gene expression profiling by microarray analysis have been investigated in various cancers (Inazawa *et al*, 2004; Ishkanian *et al*, 2004). Several oncogenes on the gained loci were identified in BC in the previous array-CGH studies; for example, *CCND1*, a member of cyclinD1, *FGF3/4*, fibroblast growth factors and *EMS1*, a regulator of cell adhesion are located on 11q13 (Zaharieva *et al*, 2003); *E2F3*, a transcription factor positively regulating cell cycle, is located on 6p22 (Hurst *et al*, 2008); and *KLK5*, a serine protease involved in cancer progression, is located on 19q13.3 (Shinoda *et al*, 2007). However, the large number of candidate genes identified by array-CGH analysis can make it difficult for researchers to identify the crucial onco-related ones. Although differential gene expression demonstrated by microarray-defined probes can be related to numerical or structural chromosomal alterations, it is unclear if such changes are also clustered in distinct chromosomes or genomic regions and whether chromosomal alterations always reflect gene expression changes. To address this issue, we used a high-resolution array containing about 244 000 probes, which enabled exhaustive detection of chromosomal gain or loss with a median spacing of 7.4 kb of cording regions. We found chromosomal copy number gains on chromosome arms 6p, 8q, 9q, 11q, 12q, 16p, and 20q and losses on chromosome arms 4p, 4q, 10p, 19p, and 21q in the five BC cell lines. Many regions showing copy number alteration matched those identified in the previous BC studies. We combined array-CGH data with mRNA expression data from oligo-microarray data analysis using five BC cell lines and 14 clinical BC samples (Kawakami *et al*, 2006). By using three different genome-wide screenings in BC, we readily identified a new target gene, *LY6K*, located at 8q24.3, which has not been identified in array-CGH research of BC. We found marked copy number gains in most of the chromosome 8q24.3 region. Several oncogenes including *MYC*, *PVT1*, *DDEF1*, *PTK2*, *GML*, and *BOP1* are located on this region, where the *LY6K* gene is closely located (Mahdy *et al*, 2001; Saramäki *et al*, 2006). Our profile showed that these oncogenes were upregulated more than two-fold in the BC cell lines (Table 3). These results suggest that the copy number gain of chromosome 8q24.3 has an important role in cancer development through overexpression of the oncogenes encoded in the region. Thus, gene profiling based on our multiple genome-wide screening enable the use of strategies for finding novel oncogenes in human BC.

Several investigations found overexpression of LY6K in several human malignancies, such as head and neck squamous cell carcinomas and breast, lung, and oesophageal cancers (de Nooij-van Dalen *et al*, 2003; Scanlan *et al*, 2004; Lee *et al*, 2006; Ishikawa *et al*, 2007; Choi *et al*, 2009; Kono *et al*, 2009). LY6K overexpression is associated with poorer prognosis for patients with non-small cell lung carcinomas as well as oesophageal squamous cell carcinomas (Ishikawa *et al*, 2007). The migration activity was significantly higher in *LY6K*-transfected breast cancer cell lines (Choi *et al*, 2009). We established a permanent *LY6K*-transfected BC cell line and identified its persistent oncogenic functions including cell growth and invasion activity as well as its migration activity, all of which were retrieved by si-*LY6K* transfection. These findings suggest that LY6K functions as an oncogenic molecule and contributes to the development of various cancers. LY6K is also a cancer/testis antigen. A new immunotherapy using established cytotoxic T lymphocyte targeting LY6K was studied in lung and oesophageal cancer cell lines (Ishikawa *et al*, 2007; Kono *et al*, 2009), meaning that LY6K is a promising target for BC immunotherapy. The other members of the LY6 family genes are mostly located at 8q24.3 (*LY6D*, *LY6E*, *LY6H*, *SLURP1*, *LYPD2*, *LYNX1*, and *GML*). The functional roles of these genes are not completely understood. Previous studies demonstrated that *LY6D* is highly expressed in colorectal cancer (Reichling *et al*, 2005) and that also *LY6E* is in pancreatic cancer stem cells (Gou *et al*, 2007). *LY6E* and *GML* as well as *LY6K* were consistently among the top 40 upregulated genes in our profile. Thus, the LY6 family genes may have an oncogenic function in cooperation. However, our profile also showed that some of the LY6 family genes were not upregulated even though they are located on 8q24.3, wherein predominantly gained loci were located in the CGH array study. These results indicate that another epigenetic pathway might repress the mRNA expression of these genes. Further studies are necessary to elucidate whether LY6K expression in clinical specimen is actually regulated by the gained locus of 8q24.3.

The LY6 family members are assumed to have functions related to cell signalling and/or cell adhesion (de Nooij-van Dalen *et al*, 2003; Lee *et al*, 2006; Choi *et al*, 2009) although the precise role of LY6K in carcinogenesis is still unknown. To gain further insight into which genes are affected by *LY6K* gene expression, we performed gene expression analysis of the *LY6K* transfectant. The functional annotations of the upregulated genes after *LY6K* transfection were distributed among nine categories including cell cycle, transcription, and signal transduction. Several clusters of genes in our profile are oncogenic molecules contributing to cancer development, for example, *E2F* genes (Hurst *et al*, 2008), insulin-like growth factors (Huszar *et al*, 2009), cell division cycle genes (Chen *et al*, 2006), *α*-/*β*-tubulines (Canta *et al*, 2009), and zinc finger proteins (Gommans *et al*, 2005; Supplementary Table SIII). These results suggest that *LY6K* promotes and activates cell-cycle-related genes in BC. Further investigation is necessary to test this hypothesis. In our cohort, there was no significant relationship between the *LY6K* mRNA expression and clinicopathological parameters. Regional epigenetic silencing and activation of multiple genes unrelated to chromosomal alterations may affect the pathological parameters of individual tumours. More precise studies with various experiments are needed to explain these phenomena.

In summary, our studies firstly demonstrated that *LY6K* gene may have an oncogenic activity in human BC and chromosomal gain locus of 8q24.3 where *LY6K* gene harbours may have a critical role for BC development. We conducted experiments to clarify the gain and loss of functions using a stable *LY6K*-transfected BC cell line and found that LY6K might have an oncogenic function, suggesting that it is a promising candidate for molecular targeting of human BC.

## Figures and Tables

**Table 1:** Patient characteristics

*Bladder cancer*	
Total number	91
Median age (range)	74 (46–100) years
Stage	
pTa	20
pT1	27
pT2	20
pT3	6
pT4	4
Unknown	14
Grade	
G1	7
G2	41
G3	31
Unknown	12
	
*Normal bladder epithelium*	
Total number	37
Median age (range)	68 (32–77) years

**Table 2:** Highly gained loci in BC cell lines

		**Location**					**Cell lines with gained loci**	
**Chromosome**	**Arm**	**Start**	**End**	**Size**	**No. of probes**	***P*-value**	**No.**	**Name**
Chr1	p36.13	16 443 872	16 570 594	126 723	18	0.0052	4	J82, T24, KK47, BOY
Chr1	p36.11	26 486 288	26 582 083	95 796	15	0.0055	4	T24, BOY, KK47, J82
Chr5	q35.3	179 048 014	179 182 725	134 712	18	0.0025	4	BOY, KK47, T24, UMUC
Chr6	p22.1	29 854 670	29 923 588	68 919	9	0.0075	4	UMUC, BOY, KK47, J82
Chr6	p21.33-p21.32	32 450 499	32 450 899	401	1	0.0012	5	KK47, UMUC, T24, BOY, J82
Chr8	q13.1	66 942 022	67 492 376	550 355	52	0.0024	4	T24, BOY, UMUC, J82
Chr8	q23.3-q24.3	115 977 013	146 294 242	30 317 230	2404	0.0009	4	UMUC, J82, KK47, BOY
Chr8	q24.3	141 934 794	146 151 558	4 216 765	496	0.0019	5	T24, UMUC, J82, BOY, KK47
Chr9	p13.3-p13.2	35 863 145	36 917 314	1054 170	105	0.0030	4	J82, KK47, UMUC, T24
Chr9	q32	116 756 726	117 059 739	303 014	45	0.0052	4	BOY, KK47, T24, J82
Chr9	q33.3-q34.3	129 659 306	140 696 609	11 037 304	1379	0.0074	4	BOY, T24, J82, KK47
Chr9	q34.13	134 348 656	135 612 832	1 264 177	145	0.0069	5	UMUC, BOY, T24, J82, KK47
Chr11	p13	33 673 844	33 768 828	94 985	13	0.0083	4	KK47, J82, UMUC, BOY
Chr11	q13.1	65 200 770	65 338 587	137 818	18	0.0011	5	UMUC, BOY, J82, T24, KK47
Chr11	q13.2-q13.4	66 619 419	74 488 807	7 869 389	807	0.0012	5	BOY, J82, T24, KK47, UMUC
Chr11	q13.3-q14.1	70 270 969	78 189 879	7 918 911	847	0.0050	5	BOY, J82, T24, UMUC, KK47
Chr12	q13.11-q13.2	46 756 675	56 434 303	9 677 629	1216	0.0013	4	T24, UMUC, BOY, KK47
Chr12	q13.12	50 421 056	50 642 687	221 632	30	0.0078	5	J82, UMUC, T24, KK47, BOY
Chr12	q13.13	53 661 113	53 814 590	153 478	25	0.0087	5	J82, UMUC, T24, KK47, BOY
Chr16	p13.3	2 880 932	2 923 782	42 851	10	0.0051	5	BOY, T24, J82, KK47, UMUC
Chr17	q25.2	74 890 765	74 958 581	67 817	7	0.0088	4	KK47, J82, T24, BOY
Chr20	q11.21-q13.33	29 833 409	62 908 815	33 075 407	3101	0.0027	5	BOY, UMUC, KK47, T24, J82
ChrX	p22.2-p22.12	16 205 283	20 103 495	3 898 213	377	0.0070	4	UMUC, J82, BOY, KK47
ChrX	q11.1-q28	62 707 611	152 734 906	90 027 296	6184	0.0061	4	KK47, UMUC, J82, BOY

Abbreviations: BC=bladder cancer; Chr=chromosome.

**Table 3:** Frequently upregulated genes on chromosome 8q24.3 in bladder cancer cell lines

			**Fold change (relative to normal bladder)**					
**Gene ID**	**Symbol**	**Gene name**	**BOY**	**J82**	**KK47**	**T24**	**UMUC**	**Average**
54742	*LY6K*	Lymphocyte antigen 6 complex, locus K	40.92	34.89	41.53	20.08	5.70	28.62
8629	*JRK*	Jerky homolog (mouse)	27.01	7.69	32.34	4.28	6.09	15.48
9401	*RECQL4*	RecQ protein-like 4	19.39	12.65	16.89	9.96	10.47	13.87
286077	*FAM83H*	Family with sequence similarity 83, member H	9.91	5.43	48.10	4.50	0.22	13.63
23246	*BOP1*	Block of proliferation 1	19.73	6.17	16.23	6.16	11.67	11.99
65263	*PYCRL*	Pyrroline-5-carboxylate reductase-like	18.65	8.13	15.94	5.00	9.25	11.39
80728	*KIAA1688*	KIAA1688 protein	14.73	8.10	18.85	6.34	6.88	10.98
4796	*NFKBIL2*	Nuclear factor of *κ* light polypeptide gene enhancer in B-cells inhibitor-like 2	16.07	5.28	13.46	6.83	10.47	10.42
286075	*ZNF707*	Zinc-finger protein 707	15.16	5.27	15.58	3.47	5.93	9.08
9684	*LRRC14*	Leucine-rich repeat containing 14	10.97	9.17	17.30	5.04	2.33	8.96
54512	*EXOSC4*	Exosome component 4	13.90	4.13	13.84	4.12	6.12	8.42
113655	*MFSD3*	Major facilitator superfamily domain containing 3	9.41	6.63	11.33	3.33	6.46	7.43
51236	*C8orf30A*	Chromosome 8 open reading frame 30A	10.67	4.59	9.19	3.88	5.83	6.83
4061	*LY6E*	Lymphocyte antigen 6 complex, locus E	6.02	7.31	10.70	2.07	5.74	6.37
9831	*ZNF623*	Zinc-finger protein 623	8.57	4.81	8.27	1.79	5.91	5.87
7264	*TSTA3*	Tissue-specific transplantation antigen P35B	7.25	2.93	10.85	3.47	3.72	5.64
1537	*CYC1*	Cytochrome *c*-1	7.88	3.96	9.05	1.96	4.55	5.48
23144	*ZC3H3*	Zinc-finger CCCH-type containing 3	7.22	2.74	9.19	2.41	4.84	5.28
28991	*COMMD5*	COMM domain containing 5	9.54	4.50	6.22	2.49	3.28	5.20
84875	*PARP10*	Poly (ADP-ribose) polymerase family, member 10	6.00	6.36	7.75	3.23	2.34	5.14
22827	*PUF60*	Poly-U-binding splicing factor 60 KDa	9.97	2.56	7.85	1.95	3.04	5.08
54108	*CHRAC1*	Chromatin accessibility complex 1	7.53	2.90	8.54	2.11	4.13	5.04
27161	*EIF2C2*	Eukaryotic translation initiation factor 2C, 2	9.83	1.98	6.74	1.97	3.14	4.73
84948	*TIGD5*	Tigger transposable element derived 5	6.12	4.47	6.86	1.61	4.33	4.68
84988	*PPP1R16A*	Protein phosphatase 1, regulatory (inhibitor) subunit 16A	5.06	2.54	10.52	1.83	3.36	4.66
26233	*FBXL6*	F-box and leucine-rich repeat protein 6	6.95	2.57	7.40	2.64	3.45	4.60
2738	*GLI4*	GLI-Kruppel family member GLI4	8.28	2.60	5.15	1.90	4.65	4.52
55630	*SLC39A4*	Solute carrier family 39 (zinc transporter), member 4	10.17	3.38	2.08	3.07	0.84	3.91
6132	*RPL8*	Ribosomal protein L8	7.12	1.86	7.13	1.33	2.05	3.90
575	*BAI1*	Brain-specific angiogenesis inhibitor 1	2.50	1.52	2.20	2.53	9.78	3.71
23513	*SCRIB*	Scribbled homologue (Drosophila)	7.46	1.81	5.32	1.56	2.35	3.70
2765	*GML*	Glycosylphosphatidylinositol anchored molecule like protein	5.18	4.34	4.46	2.68	1.68	3.67
5747	*PTK2*	Protein tyrosine kinase 2	7.73	2.24	2.84	1.76	3.77	3.67
79581	*GPR172A*	G protein-coupled receptor 172A	5.08	2.83	5.33	2.04	2.93	3.64
2875	*GPT*	Glutamic-pyruvate transaminase (alanine aminotransferase)	3.20	3.36	2.28	3.01	6.15	3.60
7553	*ZNF7*	Zinc-finger protein 7	5.71	2.46	5.24	1.67	2.69	3.55
286122	*C8orf31*	Chromosome 8 open reading frame 31	4.01	1.86	7.06	1.62	2.38	3.39
286128	*ZFP41*	Zinc-finger protein 41 homolog (mouse)	6.34	3.50	3.41	2.11	1.50	3.37
81858	*SHARPIN*	SHANK-associated RH domain interactor	5.16	2.77	4.26	1.64	2.61	3.29
114822	*RHPN1*	Rhophilin, Rho GTPase-binding protein 1	5.25	1.09	5.86	2.45	1.64	3.26
1E+08	*UNQ601*	LPPA601	3.28	1.92	5.75	1.51	3.23	3.14
79943	*ZNF696*	Zinc-finger protein 696	5.60	1.52	3.45	2.17	2.91	3.13
8928	*FOXH1*	Forkhead box H1	4.56	2.32	3.38	2.13	3.24	3.13
8733	*GPAA1*	Glycosylphosphatidylinositol anchor attachment protein 1 homolog (yeast)	4.50	2.32	4.44	1.22	2.55	3.01
340390	*KIAA1875*	KIAA1875	3.59	1.93	5.25	1.80	2.23	2.96
340371	*NRBP2*	Nuclear receptor-binding protein 2	4.13	2.32	4.67	1.18	2.31	2.92
116447	*TOP1MT*	Topoisomerase (DNA) I, mitochondrial	5.50	1.15	4.17	0.97	2.56	2.87
90990	*KIFC2*	Kinesin family member C2	1.15	4.41	2.32	2.14	4.04	2.81
2907	*GRINA*	Glutamate receptor, ionotropic, *N*-methyl D-aspartate-associated protein 1 (glutamate binding)	4.50	2.51	3.57	0.73	1.52	2.57
1936	*EEF1D*	Eukaryotic translation elongation factor 1 delta (guanine nucleotide exchange protein)	3.20	1.60	4.90	1.16	1.39	2.45
8694	*DGAT1*	Diacylglycerol O-acyltransferase homolog 1 (mouse)	3.78	1.16	3.97	0.71	2.37	2.40
7564	*ZNF16*	Zinc-finger protein 16	4.34	1.23	3.58	0.86	1.97	2.40
1584	*CYP11B1*	Cytochrome P450, family 11, subfamily B, polypeptide 1	3.01	1.91	3.00	1.48	1.92	2.27
100130274	*LOC100130274*	Similar to hCG1646697	3.40	1.57	2.62	1.52	1.66	2.15
58500	*ZNF250*	Zinc-finger protein 250	3.47	1.74	2.22	1.02	1.91	2.07
203054	*ADCK5*	aarF domain containing kinase 5	2.47	2.05	3.23	1.06	1.26	2.01
								
*Other LY6 family genes*								
137797	*LYPD2*	LY6/PLAUR domain containing 2	1.75	1.56	1.90	0.91	0.57	1.34
66004	*LYNX1*	Ly6/neurotoxin 1	1.15	0.60	1.05	0.55	1.10	0.89
57152	*SLURP1*	Secreted LY6/PLAUR domain containing 1	0.80	0.51	0.72	0.16	0.44	0.53
4062	*LY6H*	Lymphocyte antigen 6 complex, locus H	0.44	0.38	0.41	0.35	0.25	0.37
8581	*LY6D*	Lymphocyte antigen 6 complex, locus D	0.15	0.09	0.18	0.07	0.10	0.12

## Supplementary Material

Supplementary Table SI

Supplementary Table SII

Supplementary Table SIII
