# Supplementary material for: LY6K is a novel molecular target in bladder cancer on basis of integrate genome-wide profiling
Source: Br J Cancer. 2010 Nov 9;104(2):376–86. doi: 10.1038/sj.bjc.6605990 (PMC3031884; doi:10.1038/sj.bjc.6605990)
Supplement: Supplementary Table SI [file 6605990x1.doc]

| **Table SI Gained loci in BC cell lines** | | | | | | | | |
| --- | --- | --- | --- | --- | --- | --- | --- | --- |
| **Chr. arm** | **Location** | | | **No. of probes** | **P-value** | **Cell lines with gained loci** | | |
|  | **Start** | **End** | **Size** |  |  | **No.** | | **Name** |
| **chr1** | **5643276** | **7019381** | **1376106** | **151** | **0.0176** | **4** | | **J82, T24, KK47, BOY** |
| **chr1** | **8688603** | **8759787** | **71185** | **10** | **0.0407** | **4** | | **J82, T24, BOY, KK47** |
| **chr1** | **16443872** | **16570594** | **126723** | **18** | **0.0052** | **4** | | **J82, T24, KK47, BOY** |
| **chr1** | **26486288** | **26582083** | **95796** | **15** | **0.0055** | **4** | | **T24, BOY, KK47, J82** |
| **chr1** | **109394694** | **110876358** | **1481665** | **185** | **0.0436** | **4** | | **J82, UMUC, T24, KK47** |
| **chr1** | **152556249** | **152586434** | **30186** | **6** | **0.0022** | **3** | | **BOY, J82, KK47** |
| **chr1** | **154857455** | **155330377** | **472923** | **97** | **0.0417** | **4** | | **T24, J82, BOY, KK47** |
| **chr1** | **200168074** | **203593971** | **3425898** | **371** | **0.0197** | **3** | | **J82, BOY, KK47** |
| **chr1** | **204494406** | **204959657** | **465252** | **43** | **0.0138** | **2** | | **BOY, KK47** |
| **chr2** | **43346239** | **43528641** | **182403** | **16** | **0.0178** | **3** | | **T24, BOY, KK47** |
| **chr2** | **61135931** | **61157984** | **22054** | **3** | **0.0453** | **2** | | **BOY, UMUC** |
| **chr2** | **101227175** | **101696680** | **469506** | **40** | **0.0056** | **2** | | **KK47, BOY** |
| **chr2** | **109170123** | **114110599** | **4940477** | **369** | **0.0405** | **4** | | **J82, KK47, T24, BOY** |
| **chr2** | **119873369** | **122766965** | **2893597** | **266** | **0.0373** | **4** | | **J82, T24, KK47, BOY** |
| **chr2** | **173928464** | **173992945** | **64482** | **9** | **0.0489** | **3** | | **J82, BOY, T24** |
| **chr3** | **48580489** | **48702936** | **122448** | **21** | **0.0186** | **2** | | **KK47, T24** |
| **chr3** | **118930895** | **120495548** | **1564654** | **159** | **0.0310** | **4** | | **KK47, BOY, UMUC, J82** |
| **chr3** | **128759339** | **129023682** | **264344** | **37** | **0.0315** | **3** | | **UMUC, BOY, KK47** |
| **chr3** | **162514334** | **162619282** | **104949** | **8** | **0.0449** | **2** | | **UMUC, J82** |
| **chr3** | **195418968** | **195447846** | **28879** | **6** | **0.0394** | **2** | | **T24, J82** |
| **chr5** | **21949** | **46115227** | **46093279** | **3188** | **0.0385** | **5** | | **UMUC, T24, BOY, J82, KK47** |
| **chr5** | **321157** | **1566710** | **1245554** | **143** | **0.0134** | **4** | | **T24, BOY, J82, KK47** |
| **chr5** | **9866561** | **11042652** | **1176092** | **91** | **0.0151** | **5** | | **UMUC, T24, BOY, J82, KK47** |
| **chr5** | **13623847** | **15009732** | **1385886** | **138** | **0.0136** | **5** | | **UMUC, T24, BOY, J82, KK47** |
| **chr5** | **31550164** | **39652389** | **8102226** | **710** | **0.0247** | **5** | | **UMUC, T24, BOY, J82, KK47** |
| **chr5** | **37671252** | **39652389** | **1981138** | **165** | **0.0252** | **5** | | **UMUC, T24, BOY, J82, KK47** |
| **chr5** | **37671252** | **44185255** | **6514004** | **504** | **0.0305** | **5** | | **UMUC, T24, BOY, J82, KK47** |
| **chr5** | **42746221** | **43480174** | **733954** | **70** | **0.0188** | **5** | | **UMUC, T24, BOY, J82, KK47** |
| **chr5** | **137164696** | **151328544** | **14163849** | **1467** | **0.0120** | **4** | | **BOY, T24, KK47, UMUC** |
| **chr5** | **140223056** | **140236540** | **13485** | **4** | **0.0381** | **3** | | **J82, BOY, UMUC** |
| **chr5** | **167564805** | **167630001** | **65197** | **9** | **0.0087** | **3** | | **T24, KK47, UMUC** |
|  |  |  |  |  |  |  | |  |
| **Table SI (continued)** | | | | | | | | |
| **chr5** | **170019791** | **170066566** | **46776** | **6** | **0.0023** | **3** | **KK47, T24, UMUC** | |
| **chr5** | **171688082** | **171938867** | **250786** | **28** | **0.0017** | **3** | **T24, KK47, UMUC** | |
| **chr5** | **175049923** | **175205983** | **156061** | **13** | **0.0225** | **3** | **T24, KK47, UMUC** | |
| **chr5** | **175881630** | **176225402** | **343773** | **44** | **0.0007** | **3** | **T24, KK47, UMUC** | |
| **chr5** | **177629894** | **178000761** | **370868** | **46** | **0.0041** | **4** | **BOY, T24, KK47, UMUC** | |
| **chr5** | **179048014** | **179182725** | **134712** | **18** | **0.0025** | **4** | **BOY, KK47, T24, UMUC** | |
| **chr6** | **293241** | **8123417** | **7830177** | **765** | **0.0313** | **4** | **BOY, UMUC, KK47, J82** | |
| **chr6** | **2619700** | **4047630** | **1427931** | **164** | **0.0170** | **4** | **UMUC, BOY, KK47, J82** | |
| **chr6** | **29553401** | **32222361** | **2668961** | **410** | **0.0323** | **5** | **T24, UMUC, BOY, KK47, J82** | |
| **chr6** | **29854670** | **29923588** | **68919** | **9** | **0.0075** | **4** | **UMUC, BOY, KK47, J82** | |
| **chr6** | **31348484** | **31847722** | **499239** | **101** | **0.0119** | **5** | **T24, UMUC, BOY, KK47, J82** | |
| **chr6** | **32450499** | **32450899** | **401** | **1** | **0.0012** | **5** | **KK47, UMUC, T24, BOY, J82** | |
| **chr6** | **78978972** | **79023469** | **44498** | **3** | **0.0003** | **2** | **T24, UMUC** | |
| **chr7** | **105331412** | **105366075** | **34664** | **5** | **0.0492** | **3** | **T24, J82, UMUC** | |
| **chr7** | **131618496** | **138376269** | **6757774** | **617** | **0.0373** | **2** | **KK47, UMUC** | |
| **chr7** | **143883829** | **143953617** | **69789** | **4** | **0.0286** | **2** | **T24, BOY** | |
| **chr7** | **148566987** | **149561796** | **994810** | **109** | **0.0426** | **4** | **J82, UMUC, KK47, T24** | |
| **chr7** | **150517380** | **151748677** | **1231298** | **162** | **0.0261** | **4** | **J82, UMUC, KK47, T24** | |
| **chr7** | **156679557** | **157488635** | **809079** | **96** | **0.0398** | **4** | **J82, UMUC, KK47, T24** | |
| **chr8** | **186277** | **43528106** | **43341830** | **3537** | **0.0168** | **2** | **UMUC, BOY** | |
| **chr8** | **11269292** | **13349263** | **2079972** | **150** | **0.0067** | **3** | **KK47, UMUC, BOY** | |
| **chr8** | **37550524** | **38825785** | **1275262** | **146** | **0.0371** | **4** | **UMUC, KK47, BOY, J82** | |
| **chr8** | **39237238** | **39386299** | **149062** | **20** | **0.0347** | **3** | **UMUC, KK47, J82** | |
| **chr8** | **39283076** | **39322885** | **39810** | **5** | **0.0414** | **3** | **UMUC, KK47, J82** | |
| **chr8** | **41366854** | **42730170** | **1363317** | **149** | **0.0286** | **2** | **J82, BOY** | |
| **chr8** | **43036458** | **47817818** | **4781361** | **39** | **0.0208** | **3** | **UMUC, J82, BOY** | |
| **chr8** | **43036458** | **43528106** | **491649** | **24** | **0.0313** | **3** | **UMUC, J82, BOY** | |
| **chr8** | **66942022** | **67492376** | **550355** | **52** | **0.0024** | **4** | **T24, BOY, UMUC, J82** | |
| **chr8** | **94841310** | **104430576** | **9589267** | **914** | **0.0036** | **3** | **UMUC, BOY, J82** | |
| **chr8** | **115977013** | **146294242** | **30317230** | **2404** | **0.0009** | **4** | **UMUC, J82, KK47, BOY** | |
| **chr8** | **120831512** | **129405873** | **8574362** | **652** | **0.0069** | **4** | **UMUC, KK47, J82, BOY** | |
| **chr8** | **121722212** | **122945995** | **1223784** | **53** | **0.0036** | **4** | **KK47, UMUC, J82, BOY** | |
| **chr8** | **123929580** | **125650529** | **1720950** | **196** | **0.0095** | **5** | **T24, UMUC, KK47, J82, BOY** | |
|  |  |  |  |  |  |  |  | |
| **Table SI (continued)** | | | | | | | | |
| **chr8** | **126635292** | **128063946** | **1428655** | **79** | **0.0039** | **4** | | **UMUC, KK47, J82, BOY** |
| **chr8** | **128130385** | **135056147** | **6925763** | **506** | **0.0007** | **4** | | **J82, UMUC, KK47, BOY** |
| **chr8** | **128130385** | **129405873** | **1275489** | **80** | **0.0019** | **4** | | **J82, UMUC, KK47, BOY** |
| **chr8** | **131927789** | **135056147** | **3128359** | **247** | **0.0005** | **4** | | **J82, UMUC, BOY, KK47** |
| **chr8** | **131927789** | **140770768** | **8842980** | **580** | **0.0007** | **4** | | **J82, UMUC, BOY, KK47** |
| **chr8** | **133914353** | **135022021** | **1107669** | **87** | **0.0004** | **4** | | **UMUC, J82, BOY, KK47** |
| **chr8** | **136253716** | **139067865** | **2814150** | **127** | **0.0020** | **4** | | **J82, BOY, UMUC, KK47** |
| **chr8** | **140297489** | **140770768** | **473280** | **32** | **0.0009** | **4** | | **J82, UMUC, BOY, KK47** |
| **chr8** | **140297489** | **141762465** | **1464977** | **153** | **0.0011** | **4** | | **J82, UMUC, BOY, KK47** |
| **chr8** | **141241316** | **141762465** | **521150** | **67** | **0.0019** | **4** | | **J82, UMUC, KK47, BOY** |
| **chr8** | **141934794** | **146151558** | **4216765** | **496** | **0.0019** | **5** | | **T24, UMUC, J82, BOY, KK47** |
| **chr8** | **145670157** | **145719637** | **49481** | **12** | **0.0019** | **5** | | **T24, J82, UMUC, BOY, KK47** |
| **chr9** | **21929610** | **21967748** | **38139** | **5** | **0.0030** | **2** | | **J82, KK47** |
| **chr9** | **33011920** | **38459675** | **5447756** | **630** | **0.0319** | **3** | | **KK47, J82, UMUC** |
| **chr9** | **35511298** | **35842325** | **331028** | **61** | **0.0130** | **3** | | **KK47, UMUC, J82** |
| **chr9** | **35863145** | **36917314** | **1054170** | **105** | **0.0030** | **4** | | **J82, KK47, UMUC, T24** |
| **chr9** | **72293643** | **87114814** | **14821172** | **1055** | **0.0294** | **2** | | **J82, KK47** |
| **chr9** | **72293643** | **141019125** | **68725483** | **6134** | **0.0298** | **2** | | **KK47, J82** |
| **chr9** | **115472488** | **117553958** | **2081471** | **237** | **0.0105** | **4** | | **BOY, KK47, T24, J82** |
| **chr9** | **116756726** | **117059739** | **303014** | **45** | **0.0052** | **4** | | **BOY, KK47, T24, J82** |
| **chr9** | **122576975** | **125376750** | **2799776** | **278** | **0.0256** | **4** | | **BOY, T24, J82, KK47** |
| **chr9** | **124539631** | **124849924** | **310294** | **32** | **0.0157** | **4** | | **BOY, T24, J82, KK47** |
| **chr9** | **125003731** | **125376750** | **373020** | **49** | **0.0424** | **4** | | **BOY, T24, J82, KK47** |
| **chr9** | **125412525** | **127552189** | **2139665** | **245** | **0.0148** | **4** | | **BOY, T24, J82, KK47** |
| **chr9** | **128749908** | **129558107** | **808200** | **56** | **0.0220** | **4** | | **BOY, T24, J82, KK47** |
| **chr9** | **129659306** | **132878441** | **3219136** | **433** | **0.0039** | **2** | | **J82, KK47** |
| **chr9** | **129659306** | **140696609** | **11037304** | **1379** | **0.0074** | **4** | | **BOY, T24, J82, KK47** |
| **chr9** | **130475931** | **131205886** | **729956** | **131** | **0.0021** | **2** | | **J82, KK47** |
| **chr9** | **134348656** | **135612832** | **1264177** | **145** | **0.0069** | **5** | | **UMUC, BOY, T24, J82, KK47** |
| **chr9** | **139574225** | **139671575** | **97351** | **19** | **0.0039** | **5** | | **UMUC, BOY, T24, J82, KK47** |
| **chr10** | **114298037** | **114745557** | **447521** | **43** | **0.0145** | **2** | | **KK47, UMUC** |
| **chr11** | **766288** | **774509** | **8222** | **3** | **0.0102** | **5** | | **UMUC, BOY, T24, J82, KK47** |
| **chr11** | **2899703** | **3314066** | **414364** | **41** | **0.0039** | **2** | | **KK47, J82** |
|  |  |  |  |  |  |  | |  |
| **Table SI (continued)** | | | | | | | | |
| **chr11** | **2899703** | **13408265** | **10508563** | **1144** | **0.0115** | **2** | **J82, KK47** | |
| **chr11** | **31696426** | **31870863** | **174438** | **20** | **0.0383** | **2** | **J82, KK47** | |
| **chr11** | **33673844** | **33768828** | **94985** | **13** | **0.0083** | **4** | **KK47, J82, UMUC, BOY** | |
| **chr11** | **44762723** | **47159388** | **2396666** | **234** | **0.0001** | **2** | **KK47, UMUC** | |
| **chr11** | **45161369** | **49840725** | **4679357** | **409** | **0.0178** | **5** | **BOY, UMUC, T24, J82, KK47** | |
| **chr11** | **45386210** | **45727770** | **341561** | **27** | **0.0171** | **4** | **BOY, J82, KK47, UMUC** | |
| **chr11** | **46957861** | **47159388** | **201528** | **21** | **0.0161** | **5** | **BOY, J82, T24, UMUC, KK47** | |
| **chr11** | **46957861** | **48239570** | **1281710** | **154** | **0.0305** | **4** | **BOY, J82, T24, KK47** | |
| **chr11** | **55416598** | **60487149** | **5070552** | **606** | **0.0225** | **3** | **T24, J82, UMUC** | |
| **chr11** | **61768038** | **62201558** | **433521** | **46** | **0.0152** | **4** | **UMUC, T24, J82, KK47** | |
| **chr11** | **65200770** | **65338587** | **137818** | **18** | **0.0011** | **5** | **UMUC, BOY, J82, T24, KK47** | |
| **chr11** | **65625830** | **65684484** | **58655** | **14** | **0.0018** | **5** | **UMUC, KK47, J82, T24, BOY** | |
| **chr11** | **66619419** | **74488807** | **7869389** | **807** | **0.0012** | **5** | **BOY, J82, T24, KK47, UMUC** | |
| **chr11** | **66712636** | **66856416** | **143781** | **21** | **0.0009** | **5** | **BOY, T24, J82, KK47, UMUC** | |
| **chr11** | **68191211** | **70588700** | **2397490** | **217** | **0.0048** | **5** | **T24, J82, KK47, UMUC, BOY** | |
| **chr11** | **68305048** | **68516240** | **211193** | **24** | **0.0225** | **5** | **KK47, T24, J82, BOY, UMUC** | |
| **chr11** | **70270969** | **70588700** | **317732** | **32** | **0.0035** | **5** | **T24, J82, UMUC, KK47, BOY** | |
| **chr11** | **70270969** | **78189879** | **7918911** | **847** | **0.0050** | **5** | **BOY, J82, T24, UMUC, KK47** | |
| **chr11** | **70609813** | **71294510** | **684698** | **73** | **0.0049** | **2** | **UMUC, KK47** | |
| **chr11** | **71938640** | **72415053** | **476414** | **53** | **0.0055** | **5** | **BOY, J82, T24, UMUC, KK47** | |
| **chr11** | **77335726** | **78053001** | **717276** | **86** | **0.0056** | **5** | **BOY, J82, UMUC, T24, KK47** | |
| **chr11** | **82305391** | **82501005** | **195615** | **17** | **0.0378** | **4** | **BOY, UMUC, T24, KK47** | |
| **chr11** | **101412692** | **102815482** | **1402791** | **143** | **0.0268** | **4** | **KK47, BOY, UMUC, T24** | |
| **chr11** | **115109069** | **115394213** | **285145** | **35** | **0.0374** | **2** | **UMUC, T24** | |
| **chr11** | **116369571** | **119596939** | **3227369** | **388** | **0.0256** | **3** | **BOY, UMUC, T24** | |
| **chr11** | **122085647** | **123036829** | **951183** | **70** | **0.0248** | **3** | **BOY, UMUC, T24** | |
| **chr11** | **123675886** | **124436037** | **760152** | **98** | **0.0389** | **2** | **UMUC, T24** | |
| **chr12** | **9637123** | **9722007** | **84885** | **4** | **0.0248** | **2** | **UMUC, KK47** | |
| **chr12** | **10113869** | **28582337** | **18468469** | **1590** | **0.0307** | **3** | **KK47, T24, UMUC** | |
| **chr12** | **28595050** | **34756350** | **6161301** | **403** | **0.0253** | **4** | **KK47, T24, UMUC, BOY** | |
| **chr12** | **41312250** | **54187597** | **12875348** | **1327** | **0.0100** | **4** | **T24, UMUC, KK47, BOY** | |
| **chr12** | **41936845** | **42835443** | **898599** | **64** | **0.0432** | **4** | **T24, UMUC, KK47, BOY** | |
| **chr12** | **43434534** | **43908641** | **474108** | **29** | **0.0241** | **3** | **UMUC, KK47, BOY** | |
|  |  |  |  |  |  |  |  | |
| **Table SI (continued)** | | | | | | | | |
| **chr12** | **44199245** | **48520588** | **4321344** | **359** | **0.0338** | **4** | **T24, UMUC, KK47, BOY** | |
| **chr12** | **46756675** | **56434303** | **9677629** | **1216** | **0.0013** | **4** | **T24, UMUC, BOY, KK47** | |
| **chr12** | **46756675** | **48520588** | **1763914** | **136** | **0.0128** | **4** | **T24, UMUC, KK47, BOY** | |
| **chr12** | **47080272** | **47863088** | **782817** | **49** | **0.0107** | **4** | **T24, UMUC, KK47, BOY** | |
| **chr12** | **49059252** | **50071104** | **1011853** | **152** | **0.0006** | **4** | **UMUC, T24, BOY, KK47** | |
| **chr12** | **50421056** | **50642687** | **221632** | **30** | **0.0078** | **5** | **J82, UMUC, T24, KK47, BOY** | |
| **chr12** | **50827101** | **51184017** | **356917** | **44** | **0.0034** | **4** | **T24, UMUC, KK47, BOY** | |
| **chr12** | **53661113** | **53814590** | **153478** | **25** | **0.0087** | **5** | **J82, UMUC, T24, KK47, BOY** | |
| **chr12** | **108913102** | **109185919** | **272818** | **36** | **0.0354** | **4** | **J82, UMUC, BOY, KK47** | |
| **chr12** | **116725385** | **117017052** | **291668** | **15** | **0.0278** | **4** | **UMUC, J82, BOY, KK47** | |
| **chr13** | **57760278** | **57775382** | **15105** | **3** | **0.0000** | **2** | **UMUC, J82** | |
| **chr13** | **94810948** | **95363290** | **552343** | **70** | **0.0323** | **3** | **J82, BOY, UMUC** | |
| **chr13** | **113230336** | **115105960** | **1875625** | **223** | **0.0091** | **3** | **UMUC, J82, BOY** | |
| **chr14** | **89358161** | **89563403** | **205243** | **9** | **0.0383** | **5** | **T24, J82, UMUC, BOY, KK47** | |
| **chr16** | **518628** | **792399** | **273772** | **46** | **0.0282** | **3** | **J82, T24, KK47** | |
| **chr16** | **2880932** | **2923782** | **42851** | **10** | **0.0051** | **5** | **BOY, T24, J82, KK47, UMUC** | |
| **chr16** | **46500541** | **90163270** | **43662730** | **3705** | **0.0438** | **4** | **J82, KK47, T24, BOY** | |
| **chr16** | **46617215** | **49596192** | **2978978** | **243** | **0.0071** | **2** | **T24, BOY** | |
| **chr16** | **56346891** | **58992091** | **2645201** | **321** | **0.0204** | **4** | **J82, KK47, T24, BOY** | |
| **chr16** | **66343735** | **70823754** | **4480020** | **560** | **0.0183** | **4** | **J82, KK47, T24, BOY** | |
| **chr16** | **74472328** | **90148534** | **15676207** | **1485** | **0.0495** | **4** | **J82, KK47, T24, BOY** | |
| **chr16** | **74485806** | **75740228** | **1254423** | **148** | **0.0298** | **4** | **J82, KK47, T24, BOY** | |
| **chr16** | **83797209** | **90067358** | **6270150** | **671** | **0.0151** | **4** | **J82, KK47, T24, BOY** | |
| **chr16** | **83842579** | **84014886** | **172308** | **23** | **0.0082** | **2** | **BOY, KK47** | |
| **chr16** | **84511440** | **86024013** | **1512574** | **154** | **0.0134** | **4** | **J82, KK47, T24, BOY** | |
| **chr16** | **87183529** | **90111408** | **2927880** | **339** | **0.0024** | **2** | **T24, BOY** | |
| **chr17** | **644141** | **8377716** | **7733576** | **978** | **0.0351** | **4** | **J82, UMUC, KK47, T24** | |
| **chr17** | **30847522** | **30932296** | **84775** | **10** | **0.0416** | **4** | **J82, BOY, T24, KK47** | |
| **chr17** | **34437275** | **34480651** | **43377** | **5** | **0.0215** | **3** | **BOY, UMUC, T24** | |
| **chr17** | **39579539** | **81109925** | **41530387** | **4032** | **0.0430** | **4** | **KK47, J82, T24, BOY** | |
| **chr17** | **70557536** | **70714329** | **156794** | **15** | **0.0068** | **2** | **BOY, KK47** | |
| **chr17** | **72868441** | **81081181** | **8212741** | **960** | **0.0219** | **4** | **KK47, J82, T24, BOY** | |
| **chr17** | **74890765** | **74958581** | **67817** | **7** | **0.0088** | **4** | **KK47, J82, T24, BOY** | |
|  |  |  |  |  |  |  |  | |
| **Table SI (continued)** | | | | | | | | |
| **chr19** | **56645060** | **56668915** | **23856** | **3** | **0.0081** | **3** | | **UMUC, KK47, T24** |
| **chr19** | **56680305** | **59063654** | **2383350** | **299** | **0.0327** | **2** | | **UMUC, T24** |
| **chr20** | **198636** | **4773861** | **4575226** | **532** | **0.0308** | **2** | | **T24, UMUC** |
| **chr20** | **1558179** | **1581099** | **22921** | **3** | **0.0384** | **2** | | **BOY, T24** |
| **chr20** | **29833409** | **42985618** | **13152210** | **1272** | **0.0035** | **3** | | **T24, KK47, J82** |
| **chr20** | **29833409** | **62908815** | **33075407** | **3101** | **0.0027** | **5** | | **BOY, UMUC, KK47, T24, J82** |
| **chr20** | **30203568** | **33449035** | **3245468** | **372** | **0.0088** | **5** | | **UMUC, BOY, T24, KK47, J82** |
| **chr20** | **32603192** | **32769798** | **166607** | **16** | **0.0020** | **5** | | **UMUC, BOY, T24, KK47, J82** |
| **chr20** | **33684094** | **35943606** | **2259513** | **264** | **0.0017** | **3** | | **KK47, T24, J82** |
| **chr20** | **34487143** | **34702844** | **215702** | **26** | **0.0094** | **5** | | **UMUC, BOY, KK47, T24, J82** |
| **chr20** | **40408689** | **41714132** | **1305444** | **125** | **0.0050** | **3** | | **T24, KK47, J82** |
| **chr20** | **44512097** | **44741222** | **229126** | **40** | **0.0016** | **5** | | **BOY, KK47, UMUC, T24, J82** |
| **chr20** | **46039248** | **51907511** | **5868264** | **459** | **0.0148** | **5** | | **BOY, KK47, UMUC, T24, J82** |
| **chr20** | **46063659** | **47393781** | **1330123** | **85** | **0.0153** | **5** | | **BOY, KK47, UMUC, T24, J82** |
| **chr20** | **47588672** | **49527727** | **1939056** | **190** | **0.0122** | **5** | | **KK47, BOY, UMUC, T24, J82** |
| **chr20** | **50706736** | **60355145** | **9648410** | **729** | **0.0053** | **5** | | **BOY, KK47, UMUC, J82, T24** |
| **chr20** | **50706736** | **51907511** | **1200776** | **79** | **0.0310** | **5** | | **BOY, KK47, UMUC, T24, J82** |
| **chr20** | **51717872** | **51907511** | **189640** | **22** | **0.0377** | **5** | | **BOY, KK47, UMUC, T24, J82** |
| **chr20** | **60263762** | **60355145** | **91384** | **12** | **0.0016** | **5** | | **BOY, KK47, UMUC, T24, J82** |
| **chr20** | **60606230** | **62860810** | **2254581** | **322** | **0.0009** | **5** | | **BOY, KK47, UMUC, J82, T24** |
| **chr20** | **62860410** | **62908815** | **48406** | **7** | **0.0082** | **4** | | **KK47, BOY, UMUC, T24** |
| **chr20** | **62860410** | **62860810** | **401** | **1** | **0.0305** | **4** | | **KK47, BOY, UMUC, T24** |
| **chrX** | **2700116** | **4062890** | **1362775** | **142** | **0.0129** | **2** | | **BOY, J82** |
| **chrX** | **2700116** | **62693926** | **59993811** | **4495** | **0.0038** | **3** | | **J82, BOY, KK47** |
| **chrX** | **2741093** | **2778689** | **37597** | **5** | **0.0181** | **2** | | **BOY, J82** |
| **chrX** | **2908269** | **3099528** | **191260** | **20** | **0.0097** | **2** | | **J82, BOY** |
| **chrX** | **3162531** | **3186460** | **23930** | **3** | **0.0092** | **3** | | **UMUC, BOY, J82** |
| **chrX** | **3205306** | **3496155** | **290850** | **26** | **0.0271** | **2** | | **BOY, J82** |
| **chrX** | **3572651** | **3608586** | **35936** | **5** | **0.0101** | **2** | | **J82, BOY** |
| **chrX** | **3858050** | **3910472** | **52423** | **14** | **0.0316** | **2** | | **BOY, J82** |
| **chrX** | **3915598** | **3921207** | **5610** | **3** | **0.0035** | **3** | | **J82, UMUC, BOY** |
| **chrX** | **4078536** | **7006705** | **2928170** | **193** | **0.0042** | **2** | | **J82, BOY** |
| **chrX** | **6754942** | **6996923** | **241982** | **15** | **0.0113** | **2** | | **BOY, J82** |
|  |  |  |  |  |  |  | |  |
| **Table SI (continued)** | | | | | | | | |
| **chrX** | **6754942** | **6872470** | **117529** | **4** | **0.0438** | **2** | | **J82, BOY** |
| **chrX** | **6921354** | **6963040** | **41687** | **3** | **0.0027** | **2** | | **BOY, J82** |
| **chrX** | **7177244** | **7329349** | **152106** | **16** | **0.0393** | **3** | | **BOY, J82, KK47** |
| **chrX** | **10715687** | **12685465** | **1969779** | **202** | **0.0083** | **3** | | **BOY, J82, KK47** |
| **chrX** | **16205283** | **20103495** | **3898213** | **377** | **0.0070** | **4** | | **UMUC, J82, BOY, KK47** |
| **chrX** | **16716976** | **16870325** | **153350** | **19** | **0.0049** | **4** | | **J82, KK47, BOY, UMUC** |
| **chrX** | **23330954** | **24623191** | **1292238** | **123** | **0.0054** | **3** | | **J82, BOY, KK47** |
| **chrX** | **25398096** | **29992854** | **4594759** | **320** | **0.0302** | **4** | | **UMUC, J82, BOY, KK47** |
| **chrX** | **31014775** | **31729873** | **715099** | **82** | **0.0203** | **3** | | **J82, BOY, KK47** |
| **chrX** | **31654703** | **31729873** | **75171** | **10** | **0.0364** | **4** | | **UMUC, J82, BOY, KK47** |
| **chrX** | **33146413** | **57931902** | **24785490** | **1872** | **0.0077** | **3** | | **J82, BOY, KK47** |
| **chrX** | **39913059** | **41437948** | **1524890** | **123** | **0.0033** | **3** | | **J82, BOY, KK47** |
| **chrX** | **45298691** | **49627415** | **4328725** | **441** | **0.0037** | **3** | | **J82, BOY, KK47** |
| **chrX** | **45298691** | **46699336** | **1400646** | **94** | **0.0039** | **3** | | **J82, BOY, KK47** |
| **chrX** | **47422052** | **49134352** | **1712301** | **238** | **0.0032** | **3** | | **J82, BOY, KK47** |
| **chrX** | **47696281** | **48306301** | **610021** | **60** | **0.0048** | **3** | | **J82, BOY, KK47** |
| **chrX** | **52954320** | **54868530** | **1914211** | **191** | **0.0014** | **2** | | **KK47, BOY** |
| **chrX** | **53228134** | **53562651** | **334518** | **38** | **0.0008** | **3** | | **J82, KK47, BOY** |
| **chrX** | **61931489** | **62645508** | **714020** | **22** | **0.0083** | **4** | | **J82, BOY, KK47, UMUC** |
| **chrX** | **62707611** | **152734906** | **90027296** | **6184** | **0.0061** | **4** | | **KK47, UMUC, J82, BOY** |
| **chrX** | **62853475** | **72080262** | **9226788** | **748** | **0.0020** | **4** | | **J82, BOY, KK47, UMUC** |
| **chrX** | **67478490** | **71615183** | **4136694** | **411** | **0.0006** | **3** | | **BOY, UMUC, KK47** |
| **chrX** | **68798349** | **69083867** | **285519** | **31** | **0.0068** | **4** | | **J82, BOY, KK47, UMUC** |
| **chrX** | **78576663** | **99623656** | **21046994** | **997** | **0.0153** | **3** | | **J82, BOY, UMUC** |
| **chrX** | **99845367** | **100759914** | **914548** | **117** | **0.0293** | **2** | | **J82, BOY** |
| **chrX** | **100740892** | **103829721** | **3088830** | **292** | **0.0325** | **3** | | **J82, BOY, UMUC** |
| **chrX** | **102949266** | **103003234** | **53969** | **11** | **0.0444** | **3** | | **J82, BOY, UMUC** |
| **chrX** | **103015149** | **103821400** | **806252** | **74** | **0.0059** | **3** | | **J82, BOY, UMUC** |
| **chrX** | **103893449** | **104126553** | **233105** | **27** | **0.0159** | **3** | | **BOY, J82, UMUC** |
| **chrX** | **103952661** | **104048222** | **95562** | **11** | **0.0133** | **3** | | **BOY, J82, UMUC** |
| **chrX** | **104077030** | **105401871** | **1324842** | **144** | **0.0181** | **2** | | **BOY, J82** |
| **chrX** | **104077030** | **104126553** | **49524** | **7** | **0.0252** | **3** | | **BOY, J82, UMUC** |
| **chrX** | **104491825** | **104649726** | **157902** | **20** | **0.0228** | **2** | | **BOY, J82** |
|  |  |  |  |  |  |  | |  |
| **Table SI (continued)** | | | | | | | | |
| **chrX** | **105912148** | **107316610** | **1404463** | **148** | **0.0221** | **3** | | **UMUC, J82, BOY** |
| **chrX** | **108619063** | **110095155** | **1476093** | **135** | **0.0260** | **2** | | **J82, BOY** |
| **chrX** | **108638350** | **108691557** | **53208** | **7** | **0.0053** | **2** | | **J82, BOY** |
| **chrX** | **108873925** | **108960552** | **86628** | **12** | **0.0212** | **2** | | **J82, BOY** |
| **chrX** | **109084548** | **109325957** | **241410** | **14** | **0.0115** | **2** | | **J82, BOY** |
| **chrX** | **109536722** | **109593523** | **56802** | **7** | **0.0443** | **2** | | **J82, BOY** |
| **chrX** | **109622421** | **109788732** | **166312** | **19** | **0.0308** | **2** | | **J82, BOY** |
| **chrX** | **109918423** | **109944106** | **25684** | **4** | **0.0169** | **2** | | **J82, BOY** |
| **chrX** | **117517883** | **120121325** | **2603443** | **260** | **0.0370** | **2** | | **J82, BOY** |
| **chrX** | **123646387** | **127358573** | **3712187** | **186** | **0.0497** | **4** | | **UMUC, J82, BOY, KK47** |
| **chrX** | **128666963** | **129539152** | **872190** | **99** | **0.0031** | **3** | | **J82, BOY, KK47** |
| **chrX** | **133569532** | **136294872** | **2725341** | **287** | **0.0056** | **3** | | **J82, BOY, KK47** |
| **chrX** | **140690951** | **140801155** | **110205** | **10** | **0.0082** | **3** | | **BOY, UMUC, J82** |
| **chrX** | **140789037** | **140801155** | **12119** | **4** | **0.0003** | **2** | | **BOY, UMUC** |
| **chrX** | **140789037** | **140868944** | **79908** | **7** | **0.0141** | **3** | | **UMUC, BOY, J82** |
| **chrX** | **144586325** | **148465315** | **3878991** | **240** | **0.0139** | **2** | | **KK47, UMUC** |
| **chrX** | **144586325** | **154929420** | **10343096** | **927** | **0.0017** | **4** | | **J82, BOY, KK47, UMUC** |
| **chrX** | **148575015** | **150153807** | **1578793** | **141** | **0.0011** | **4** | | **J82, BOY, KK47, UMUC** |
| **chrX** | **151730516** | **152734906** | **1004391** | **86** | **0.0004** | **4** | | **J82, BOY, UMUC, KK47** |
| **chrX** | **152515334** | **152734906** | **219573** | **23** | **0.0001** | **4** | | **J82, BOY, UMUC, KK47** |
| **chrX** | **152559905** | **154251123** | **1691219** | **240** | **0.0001** | **4** | | **J82, BOY, UMUC, KK47** |
| **chrX** | **153902559** | **154425825** | **523267** | **64** | **0.0006** | **4** | | **J82, BOY, KK47, UMUC** |
| **chrY** | **7679871** | **7729911** | **50041** | **10** | **0.0138** | **2** | | **J82, T24** |
| **chrY** | **7858699** | **9901455** | **2042757** | **180** | **0.0398** | **2** | | **T24, J82** |
| **chrY** | **7954895** | **8098434** | **143540** | **23** | **0.0170** | **2** | | **T24, J82** |
| **chrY** | **8170556** | **8227173** | **56618** | **11** | **0.0303** | **2** | | **T24, J82** |
| **chrY** | **8554017** | **8592893** | **38877** | **5** | **0.0072** | **2** | | **J82, T24** |
| **chrY** | **8809490** | **8875334** | **65845** | **5** | **0.0202** | **2** | | **J82, T24** |
| **chrY** | **9386972** | **9650335** | **263364** | **10** | **0.0184** | **2** | | **T24, J82** |
| **chrY** | **14108063** | **14128187** | **20125** | **3** | **0.0383** | **2** | | **J82, T24** |
| **chrY** | **14777114** | **14918377** | **141264** | **24** | **0.0335** | **2** | | **T24, J82** |
| **chrY** | **14945057** | **14971968** | **26912** | **6** | **0.0440** | **2** | | **T24, J82** |
| **chrY** | **16786904** | **16835577** | **48674** | **11** | **0.0008** | **2** | | **T24, J82** |
|  |  |  |  |  |  |  | |  |
| **Table SI (continued)** | | | | | | | | |
| **chrY** | **17197713** | **17402195** | **204483** | **11** | **0.0187** | **2** | | **J82, T24** |
| **chrY** | **21728154** | **21742027** | **13874** | **5** | **0.0400** | **2** | | **T24, J82** |
| **chrY** | **21765894** | **21801867** | **35974** | **5** | **0.0349** | **2** | | **T24, J82** |
| **chrY** | **23331457** | **23384443** | **52987** | **4** | **0.0024** | **2** | | **J82, T24** |
| **chrY** | **23545233** | **23550672** | **5440** | **3** | **0.0137** | **2** | | **J82, T24** |
| **chrY** | **23766153** | **23962272** | **196120** | **8** | **0.0449** | **2** | | **J82, T24** |
| **chrY** | **23971871** | **23986608** | **14738** | **3** | **0.0257** | **2** | | **T24, J82** |
| **chrY** | **24441392** | **28562993** | **4121602** | **32** | **0.0370** | **2** | | **T24, J82** |
| **chrY** | **24498754** | **24514534** | **15781** | **5** | **0.0062** | **2** | | **J82, T24** |
