# Supplementary material for: LY6K is a novel molecular target in bladder cancer on basis of integrate genome-wide profiling
Source: Br J Cancer. 2010 Nov 9;104(2):376–86. doi: 10.1038/sj.bjc.6605990 (PMC3031884; doi:10.1038/sj.bjc.6605990)
Supplement: Supplementary Table SII [file 6605990x2.doc]

| **Table SII Lost loci in BC cell lines** | | | | | | | |
| --- | --- | --- | --- | --- | --- | --- | --- |
| **Chr. arm** | **Location** | | | **No. of probes** | **P-value** | **Cell lines with lost loci** | |
|  | **Start** | **End** | **Size** |  |  | **No.** | **Name** |
| **chr1** | **57956263** | **57973359** | **17097** | **3** | **0.0116** | **2** | **J82, BOY** |
| **chr1** | **72075092** | **72353984** | **278893** | **31** | **0.0180** | **5** | **KK47, T24, UMUC, J82, BOY** |
| **chr1** | **72368068** | **72406593** | **38526** | **4** | **0.0344** | **5** | **UMUC, KK47, BOY, J82, T24** |
| **chr1** | **72391456** | **72406593** | **15138** | **2** | **0.0411** | **5** | **UMUC, KK47, BOY, J82, T24** |
| **chr1** | **72557761** | **72578421** | **20661** | **3** | **0.0360** | **2** | **UMUC, T24** |
| **chr1** | **72656610** | **72742240** | **85631** | **8** | **0.0484** | **5** | **UMUC, J82, BOY, T24, KK47** |
| **chr1** | **175930731** | **176170810** | **240080** | **27** | **0.0024** | **5** | **KK47, T24, UMUC, J82, BOY** |
| **chr1** | **196138563** | **199972464** | **3833902** | **300** | **0.0325** | **4** | **UMUC, T24, J82, BOY** |
| **chr1** | **197566011** | **197724383** | **158373** | **18** | **0.0379** | **4** | **BOY, UMUC, T24, J82** |
| **chr1** | **248438206** | **248531431** | **93226** | **15** | **0.0094** | **4** | **KK47, T24, J82, UMUC** |
| **chr2** | **3852394** | **8218913** | **4366520** | **218** | **0.0309** | **3** | **J82, KK47, UMUC** |
| **chr2** | **8551444** | **12130212** | **3578769** | **336** | **0.0446** | **3** | **J82, KK47, UMUC** |
| **chr2** | **12960876** | **17959474** | **4998599** | **262** | **0.0259** | **3** | **J82, KK47, UMUC** |
| **chr2** | **19545437** | **21123506** | **1578070** | **116** | **0.0441** | **3** | **J82, KK47, UMUC** |
| **chr2** | **33895822** | **36567999** | **2672178** | **98** | **0.0277** | **3** | **J82, KK47, UMUC** |
| **chr2** | **61135931** | **61157984** | **22054** | **3** | **0.0383** | **2** | **T24, J82** |
| **chr2** | **72606893** | **72998333** | **391441** | **44** | **0.0289** | **4** | **J82, T24, UMUC, BOY** |
| **chr2** | **76077939** | **84251519** | **8173581** | **457** | **0.0289** | **3** | **J82, T24, BOY** |
| **chr2** | **77639262** | **77734714** | **95453** | **8** | **0.0313** | **5** | **UMUC, J82, T24, BOY, KK47** |
| **chr2** | **77639262** | **78467081** | **827820** | **49** | **0.0234** | **4** | **UMUC, J82, T24, BOY** |
| **chr2** | **77752008** | **77919623** | **167616** | **9** | **0.0420** | **5** | **UMUC, J82, BOY, T24, KK47** |
| **chr2** | **77946399** | **78142796** | **196398** | **8** | **0.0016** | **2** | **UMUC, J82** |
| **chr2** | **78169468** | **78231420** | **61953** | **4** | **0.0492** | **4** | **UMUC, J82, T24, BOY** |
| **chr2** | **78274118** | **78467081** | **192964** | **14** | **0.0295** | **5** | **UMUC, J82, T24, BOY, KK47** |
| **chr2** | **78495478** | **78915016** | **419539** | **28** | **0.0064** | **4** | **J82, UMUC, T24, BOY** |
| **chr2** | **79094889** | **81692747** | **2597859** | **216** | **0.0333** | **3** | **J82, T24, BOY** |
| **chr2** | **132344575** | **132490096** | **145522** | **9** | **0.0334** | **4** | **KK47, J82, UMUC, T24** |
| **chr2** | **140017844** | **141102689** | **1084846** | **58** | **0.0213** | **3** | **KK47, UMUC, J82** |
| **chr2** | **141108346** | **142274839** | **1166494** | **122** | **0.0381** | **3** | **KK47, UMUC, J82** |
| **chr2** | **141416566** | **141906359** | **489794** | **52** | **0.0197** | **3** | **KK47, UMUC, J82** |
| **chr2** | **141936972** | **142274839** | **337868** | **35** | **0.0203** | **2** | **UMUC, KK47** |
|  |  |  |  |  |  |  |  |
| **Table SII (continued)** | | | | | | | |
| **chr2** | **142577834** | **142827815** | **249982** | **28** | **0.0234** | **5** | **KK47, UMUC, J82, T24, BOY** |
| **chr2** | **153514213** | **157099694** | **3585482** | **191** | **0.0211** | **3** | **KK47, UMUC, J82** |
| **chr2** | **153672995** | **154539032** | **866038** | **33** | **0.0081** | **4** | **BOY, KK47, UMUC, J82** |
| **chr2** | **154568998** | **156046154** | **1477157** | **104** | **0.0169** | **3** | **KK47, UMUC, J82** |
| **chr2** | **156079425** | **157099694** | **1020270** | **38** | **0.0074** | **4** | **BOY, KK47, UMUC, J82** |
| **chr2** | **156079425** | **166712089** | **10632665** | **834** | **0.0148** | **3** | **BOY, KK47, UMUC** |
| **chr2** | **163291526** | **166698014** | **3406489** | **264** | **0.0114** | **4** | **BOY, KK47, UMUC, J82** |
| **chr2** | **166536074** | **168921012** | **2384939** | **186** | **0.0127** | **4** | **KK47, UMUC, J82, BOY** |
| **chr2** | **166536074** | **166712089** | **176016** | **22** | **0.0127** | **4** | **BOY, KK47, UMUC, J82** |
| **chr2** | **175087421** | **175160808** | **73388** | **7** | **0.0185** | **2** | **UMUC, KK47** |
| **chr2** | **180515386** | **182167057** | **1651672** | **95** | **0.0300** | **3** | **KK47, UMUC, J82** |
| **chr2** | **184203552** | **186857226** | **2653675** | **144** | **0.0101** | **4** | **KK47, UMUC, J82, BOY** |
| **chr2** | **187884081** | **189890214** | **2006134** | **133** | **0.0414** | **3** | **BOY, KK47, J82** |
| **chr2** | **193780076** | **196913206** | **3133131** | **131** | **0.0261** | **3** | **BOY, KK47, J82** |
| **chr2** | **194325544** | **195326033** | **1000490** | **38** | **0.0091** | **3** | **J82, BOY, KK47** |
| **chr2** | **204701557** | **206354186** | **1652630** | **141** | **0.0005** | **3** | **BOY, KK47, UMUC** |
| **chr2** | **206239566** | **206429231** | **189666** | **24** | **0.0051** | **3** | **UMUC, BOY, KK47** |
| **chr2** | **206389706** | **206429231** | **39526** | **6** | **0.0034** | **4** | **UMUC, J82, KK47, BOY** |
| **chr2** | **206389706** | **207907250** | **1517545** | **138** | **0.0030** | **4** | **J82, BOY, KK47, UMUC** |
| **chr2** | **207923350** | **209153729** | **1230380** | **115** | **0.0086** | **3** | **BOY, KK47, UMUC** |
| **chr2** | **208002989** | **208425958** | **422970** | **29** | **0.0117** | **3** | **BOY, KK47, UMUC** |
| **chr2** | **211533875** | **214472808** | **2938934** | **237** | **0.0085** | **3** | **BOY, KK47, UMUC** |
| **chr2** | **215579798** | **224453255** | **8873458** | **768** | **0.0081** | **3** | **J82, KK47, UMUC** |
| **chr2** | **220752524** | **231106290** | **10353767** | **804** | **0.0229** | **3** | **J82, KK47, UMUC** |
| **chr2** | **220752524** | **224453255** | **3700732** | **215** | **0.0195** | **3** | **J82, KK47, UMUC** |
| **chr2** | **225008164** | **225132960** | **124797** | **6** | **0.0145** | **2** | **J82, UMUC** |
| **chr2** | **240115852** | **240155118** | **39267** | **5** | **0.0143** | **4** | **T24, J82, KK47, UMUC** |
| **chr3** | **63866** | **3996674** | **3932809** | **357** | **0.0057** | **3** | **BOY, J82, KK47** |
| **chr3** | **3895839** | **9772308** | **5876470** | **457** | **0.0077** | **4** | **UMUC, BOY, KK47, J82** |
| **chr3** | **3895839** | **3996674** | **100836** | **6** | **0.0052** | **4** | **UMUC, BOY, J82, KK47** |
| **chr3** | **5861392** | **7733053** | **1871662** | **140** | **0.0024** | **4** | **UMUC, BOY, KK47, J82** |
| **chr3** | **17394025** | **22743692** | **5349668** | **331** | **0.0188** | **3** | **BOY, J82, UMUC** |
| **chr3** | **34506417** | **36390196** | **1883780** | **106** | **0.0183** | **3** | **BOY, J82, UMUC** |
|  |  |  |  |  |  |  |  |
| **Table SII (continued)** | | | | | | | |
| **chr3** | **34918131** | **35719662** | **801532** | **51** | **0.0184** | **4** | **BOY, J82, UMUC, T24** |
| **chr3** | **49378210** | **49572249** | **194040** | **27** | **0.0079** | **2** | **BOY, UMUC** |
| **chr3** | **54241251** | **56359777** | **2118527** | **207** | **0.0411** | **3** | **BOY, UMUC, J82** |
| **chr3** | **54985324** | **55444687** | **459364** | **24** | **0.0437** | **3** | **BOY, J82, UMUC** |
| **chr3** | **58949111** | **68765927** | **9816817** | **802** | **0.0022** | **4** | **BOY, KK47, UMUC, J82** |
| **chr3** | **59947596** | **60201884** | **254289** | **31** | **0.0032** | **5** | **UMUC, BOY, T24, KK47, J82** |
| **chr3** | **59947596** | **60735816** | **788221** | **86** | **0.0015** | **4** | **UMUC, T24, KK47, BOY** |
| **chr3** | **60019942** | **60076877** | **56936** | **7** | **0.0079** | **5** | **BOY, UMUC, T24, KK47, J82** |
| **chr3** | **60213105** | **60256858** | **43754** | **6** | **0.0128** | **5** | **T24, UMUC, BOY, J82, KK47** |
| **chr3** | **60213105** | **60522834** | **309730** | **31** | **0.0043** | **4** | **T24, UMUC, KK47, BOY** |
| **chr3** | **60269709** | **61205369** | **935661** | **99** | **0.0033** | **5** | **KK47, UMUC, T24, BOY, J82** |
| **chr3** | **60314214** | **60356399** | **42186** | **4** | **0.0066** | **3** | **KK47, T24, UMUC** |
| **chr3** | **60528540** | **60630005** | **101466** | **12** | **0.0147** | **5** | **UMUC, KK47, T24, BOY, J82** |
| **chr3** | **60585046** | **60630005** | **44960** | **6** | **0.0120** | **5** | **UMUC, KK47, T24, BOY, J82** |
| **chr3** | **60585046** | **60703969** | **118924** | **14** | **0.0044** | **5** | **UMUC, KK47, T24, BOY, J82** |
| **chr3** | **60642527** | **60665185** | **22659** | **3** | **0.0089** | **5** | **UMUC, KK47, T24, BOY, J82** |
| **chr3** | **60735416** | **60735816** | **401** | **1** | **0.0218** | **5** | **KK47, UMUC, BOY, T24, J82** |
| **chr3** | **60735416** | **60794047** | **58632** | **7** | **0.0215** | **5** | **KK47, UMUC, BOY, T24, J82** |
| **chr3** | **60817292** | **60858859** | **41568** | **6** | **0.0294** | **5** | **KK47, BOY, J82, T24, UMUC** |
| **chr3** | **73777674** | **90166928** | **16389255** | **969** | **0.0020** | **5** | **BOY, KK47, J82, UMUC, T24** |
| **chr3** | **82112938** | **86082641** | **3969704** | **237** | **0.0018** | **5** | **BOY, KK47, UMUC, J82, T24** |
| **chr3** | **82112938** | **83913421** | **1800484** | **71** | **0.0025** | **3** | **BOY, KK47, J82** |
| **chr3** | **84959121** | **90166928** | **5207808** | **387** | **0.0027** | **5** | **BOY, T24, KK47, UMUC, J82** |
| **chr3** | **84959121** | **86082641** | **1123521** | **129** | **0.0017** | **5** | **BOY, KK47, T24, UMUC, J82** |
| **chr3** | **86719777** | **88328589** | **1608813** | **100** | **0.0030** | **4** | **BOY, T24, KK47, UMUC** |
| **chr3** | **94130367** | **97124975** | **2994609** | **155** | **0.0071** | **4** | **KK47, T24, BOY, J82** |
| **chr3** | **153215232** | **154900364** | **1685133** | **93** | **0.0265** | **2** | **KK47, T24** |
| **chr3** | **162768669** | **167816211** | **5047543** | **278** | **0.0143** | **4** | **KK47, T24, J82, BOY** |
| **chr3** | **172827781** | **176197266** | **3369486** | **271** | **0.0132** | **2** | **T24, KK47** |
| **chr3** | **177804363** | **178685570** | **881208** | **60** | **0.0153** | **2** | **T24, KK47** |
| **chr3** | **179537266** | **182721639** | **3184374** | **174** | **0.0128** | **2** | **KK47, T24** |
| **chr4** | **51213** | **49083431** | **49032219** | **3520** | **0.0032** | **5** | **UMUC, BOY, KK47, T24, J82** |
| **chr4** | **10483775** | **23377933** | **12894159** | **858** | **0.0024** | **4** | **UMUC, BOY, KK47, T24** |
|  |  |  |  |  |  |  |  |

| **Table SII (continued)** | | | | | | | |
| --- | --- | --- | --- | --- | --- | --- | --- |
| **chr4** | **10698690** | **12054442** | **1355753** | **52** | **0.0011** | **4** | **UMUC, BOY, KK47, T24** |
| **chr4** | **12148877** | **12322960** | **174084** | **7** | **0.0154** | **5** | **BOY, UMUC, KK47, T24, J82** |
| **chr4** | **13328102** | **15060194** | **1732093** | **140** | **0.0024** | **4** | **BOY, UMUC, KK47, T24** |
| **chr4** | **17575623** | **22563733** | **4988111** | **365** | **0.0018** | **4** | **UMUC, BOY, KK47, T24** |
| **chr4** | **28067672** | **36195409** | **8127738** | **309** | **0.0002** | **5** | **BOY, UMUC, KK47, J82, T24** |
| **chr4** | **28859691** | **32667741** | **3808051** | **156** | **0.0012** | **5** | **BOY, UMUC, KK47, T24, J82** |
| **chr4** | **31806006** | **32667741** | **861736** | **25** | **0.0008** | **5** | **UMUC, BOY, KK47, T24, J82** |
| **chr4** | **31806006** | **49083431** | **17277426** | **1195** | **0.0007** | **5** | **J82, UMUC, BOY, KK47, T24** |
| **chr4** | **33568879** | **36574427** | **3005549** | **128** | **0.0004** | **5** | **J82, BOY, UMUC, KK47, T24** |
| **chr4** | **42106869** | **47155136** | **5048268** | **308** | **0.0003** | **5** | **J82, UMUC, BOY, KK47, T24** |
| **chr4** | **52688901** | **109903722** | **57214822** | **4402** | **0.0008** | **5** | **UMUC, J82, BOY, KK47, T24** |
| **chr4** | **52688901** | **169076319** | **116387419** | **8285** | **0.0003** | **5** | **J82, BOY, UMUC, KK47, T24** |
| **chr4** | **53697546** | **57999876** | **4302331** | **377** | **0.0019** | **5** | **UMUC, J82, BOY, KK47, T24** |
| **chr4** | **58329124** | **58365561** | **36438** | **2** | **0.0003** | **5** | **UMUC, J82, BOY, T24, KK47** |
| **chr4** | **58618826** | **71058988** | **12440163** | **668** | **0.0004** | **5** | **J82, UMUC, BOY, KK47, T24** |
| **chr4** | **67707926** | **69223275** | **1515350** | **117** | **0.0002** | **2** | **UMUC, J82** |
| **chr4** | **68332734** | **68355222** | **22489** | **3** | **0.0077** | **4** | **KK47, J82, UMUC, BOY** |
| **chr4** | **69374940** | **69414941** | **40002** | **5** | **0.0113** | **2** | **UMUC, BOY** |
| **chr4** | **69456242** | **69483419** | **27178** | **4** | **0.0010** | **2** | **UMUC, BOY** |
| **chr4** | **73363293** | **88901732** | **15538440** | **1356** | **0.0012** | **5** | **UMUC, J82, BOY, KK47, T24** |
| **chr4** | **80085460** | **82337984** | **2252525** | **185** | **0.0005** | **5** | **J82, UMUC, KK47, BOY, T24** |
| **chr4** | **81316683** | **81637107** | **320425** | **35** | **0.0050** | **5** | **KK47, J82, UMUC, BOY, T24** |
| **chr4** | **88949341** | **89061368** | **112028** | **12** | **0.0331** | **5** | **UMUC, J82, BOY, KK47, T24** |
| **chr4** | **91185097** | **94523621** | **3338525** | **317** | **0.0003** | **5** | **UMUC, J82, BOY, KK47, T24** |
| **chr4** | **91842548** | **91926538** | **83991** | **12** | **0.0004** | **4** | **UMUC, T24, J82, BOY** |
| **chr4** | **92209718** | **92396739** | **187022** | **19** | **0.0018** | **5** | **KK47, J82, UMUC, BOY, T24** |
| **chr4** | **94224449** | **94611597** | **387149** | **41** | **0.0055** | **5** | **UMUC, J82, BOY, KK47, T24** |
| **chr4** | **95255487** | **96235768** | **980282** | **102** | **0.0009** | **5** | **J82, UMUC, BOY, KK47, T24** |
| **chr4** | **96783111** | **98822367** | **2039257** | **116** | **0.0002** | **5** | **UMUC, J82, BOY, KK47, T24** |
| **chr4** | **109668974** | **109867508** | **198535** | **25** | **0.0115** | **5** | **UMUC, J82, BOY, KK47, T24** |
| **chr4** | **115531661** | **119095348** | **3563688** | **160** | **0.0018** | **5** | **J82, BOY, KK47, T24, UMUC** |
| **chr4** | **125409843** | **127774259** | **2364417** | **102** | **0.0047** | **5** | **J82, BOY, KK47, T24, UMUC** |
| **chr4** | **130609236** | **138206201** | **7596966** | **290** | **0.0016** | **5** | **J82, BOY, KK47, T24, UMUC** |
|  |  |  |  |  |  |  |  |

| **Table SII (continued)** | | | | | | | |
| --- | --- | --- | --- | --- | --- | --- | --- |
| **chr4** | **142169548** | **143703213** | **1533666** | **108** | **0.0012** | **4** | **J82, BOY, T24, KK47** |
| **chr4** | **151264698** | **151599685** | **334988** | **43** | **0.0012** | **4** | **J82, BOY, T24, KK47** |
| **chr4** | **151873262** | **155157446** | **3284185** | **256** | **0.0040** | **5** | **J82, BOY, KK47, T24, UMUC** |
| **chr4** | **154805238** | **183135541** | **28330304** | **1831** | **0.0033** | **5** | **KK47, BOY, T24, J82, UMUC** |
| **chr4** | **154805238** | **155157446** | **352209** | **13** | **0.0012** | **5** | **BOY, J82, KK47, T24, UMUC** |
| **chr4** | **160935409** | **163855246** | **2919838** | **157** | **0.0010** | **5** | **BOY, KK47, T24, J82, UMUC** |
| **chr4** | **166938802** | **190791022** | **23852221** | **1678** | **0.0177** | **5** | **KK47, BOY, T24, J82, UMUC** |
| **chr4** | **166938802** | **169076319** | **2137518** | **123** | **0.0079** | **5** | **KK47, BOY, T24, J82, UMUC** |
| **chr4** | **171351503** | **173505291** | **2153789** | **134** | **0.0075** | **5** | **KK47, BOY, T24, J82, UMUC** |
| **chr4** | **174116520** | **174191122** | **74603** | **8** | **0.0227** | **5** | **UMUC, KK47, BOY, T24, J82** |
| **chr4** | **178712716** | **182524050** | **3811335** | **128** | **0.0092** | **5** | **KK47, BOY, T24, J82, UMUC** |
| **chr4** | **183612276** | **187542911** | **3930636** | **386** | **0.0373** | **5** | **KK47, BOY, T24, UMUC, J82** |
| **chr5** | **49559720** | **50053680** | **493961** | **31** | **0.0416** | **3** | **UMUC, KK47, J82** |
| **chr5** | **58565111** | **58597524** | **32414** | **5** | **0.0361** | **2** | **KK47, UMUC** |
| **chr5** | **128842720** | **128965743** | **123024** | **15** | **0.0320** | **2** | **BOY, J82** |
| **chr5** | **147497723** | **147678686** | **180964** | **22** | **0.0205** | **2** | **BOY, J82** |
| **chr6** | **32450499** | **32552312** | **101814** | **10** | **0.0176** | **4** | **J82, BOY, T24, KK47** |
| **chr6** | **32459204** | **32493184** | **33981** | **4** | **0.0011** | **4** | **J82, BOY, T24, KK47** |
| **chr6** | **32487224** | **32522070** | **34847** | **4** | **0.0140** | **3** | **J82, T24, BOY** |
| **chr6** | **32487224** | **32493184** | **5961** | **2** | **0.0032** | **4** | **J82, BOY, KK47, T24** |
| **chr6** | **65598494** | **70709733** | **5111240** | **282** | **0.0270** | **3** | **KK47, J82, BOY** |
| **chr6** | **114600388** | **115792907** | **1192520** | **33** | **0.0092** | **4** | **KK47, T24, J82, BOY** |
| **chr6** | **120138398** | **124636212** | **4497815** | **283** | **0.0154** | **4** | **KK47, J82, T24, BOY** |
| **chr6** | **145473668** | **147062969** | **1589302** | **134** | **0.0454** | **4** | **KK47, J82, T24, BOY** |
| **chr6** | **162020346** | **165191458** | **3171113** | **247** | **0.0195** | **4** | **KK47, T24, J82, BOY** |
| **chr7** | **44930** | **34296983** | **34252054** | **3024** | **0.0441** | **2** | **UMUC, BOY** |
| **chr7** | **7660491** | **20710918** | **13050428** | **963** | **0.0066** | **2** | **UMUC, BOY** |
| **chr7** | **13993281** | **15614615** | **1621335** | **143** | **0.0184** | **2** | **BOY, UMUC** |
| **chr7** | **48403402** | **54418422** | **6015021** | **358** | **0.0174** | **5** | **BOY, KK47, J82, UMUC, T24** |
| **chr7** | **48433008** | **50196055** | **1763048** | **142** | **0.0101** | **5** | **BOY, KK47, J82, UMUC, T24** |
| **chr7** | **51956251** | **54418422** | **2462172** | **94** | **0.0120** | **5** | **BOY, KK47, J82, UMUC, T24** |
| **chr7** | **62030465** | **64435594** | **2405130** | **125** | **0.0359** | **4** | **BOY, KK47, J82, T24** |
| **chr7** | **67148569** | **71738999** | **4590431** | **347** | **0.0361** | **4** | **BOY, KK47, T24, J82** |
|  |  |  |  |  |  |  |  |

| **Table SII (continued)** | | | | | | | |
| --- | --- | --- | --- | --- | --- | --- | --- |
| **chr7** | **69628026** | **69764483** | **136458** | **18** | **0.0153** | **5** | **UMUC, BOY, KK47, T24, J82** |
| **chr7** | **77708161** | **85947711** | **8239551** | **592** | **0.0221** | **4** | **BOY, KK47, T24, J82** |
| **chr7** | **88773110** | **90272215** | **1499106** | **102** | **0.0246** | **4** | **BOY, KK47, T24, J82** |
| **chr7** | **107771420** | **127073106** | **19301687** | **1408** | **0.0288** | **4** | **BOY, KK47, J82, T24** |
| **chr7** | **110607390** | **110754075** | **146686** | **18** | **0.0279** | **5** | **UMUC, BOY, KK47, T24, J82** |
| **chr7** | **110669425** | **110708211** | **38787** | **6** | **0.0382** | **5** | **UMUC, BOY, KK47, T24, J82** |
| **chr7** | **111840799** | **112296067** | **455269** | **46** | **0.0367** | **2** | **BOY, KK47** |
| **chr7** | **117851169** | **120175613** | **2324445** | **107** | **0.0167** | **4** | **BOY, KK47, J82, T24** |
| **chr7** | **120501191** | **120559902** | **58712** | **4** | **0.0158** | **2** | **BOY, KK47** |
| **chr8** | **2104492** | **6043400** | **3938909** | **327** | **0.0385** | **5** | **T24, J82, BOY, KK47, UMUC** |
| **chr8** | **7268619** | **7752727** | **484109** | **7** | **0.0000** | **3** | **J82, UMUC, T24** |
| **chr8** | **13603886** | **17153270** | **3549385** | **227** | **0.0467** | **4** | **T24, J82, BOY, KK47** |
| **chr9** | **162931** | **35721709** | **35558779** | **2760** | **0.0282** | **2** | **T24, BOY** |
| **chr9** | **8389016** | **8436778** | **47763** | **6** | **0.0238** | **3** | **KK47, BOY, T24** |
| **chr9** | **9053125** | **9213576** | **160452** | **15** | **0.0091** | **3** | **T24, BOY, KK47** |
| **chr9** | **9225265** | **9433788** | **208524** | **16** | **0.0374** | **4** | **T24, BOY, KK47, J82** |
| **chr9** | **9453633** | **12388040** | **2934408** | **149** | **0.0046** | **3** | **T24, BOY, KK47** |
| **chr9** | **17394682** | **17521941** | **127260** | **17** | **0.0437** | **2** | **T24, BOY** |
| **chr9** | **21957535** | **22036646** | **79112** | **14** | **0.0094** | **2** | **UMUC, BOY** |
| **chr9** | **21957535** | **21967748** | **10214** | **2** | **0.0035** | **2** | **UMUC, BOY** |
| **chr9** | **22003451** | **22009182** | **5732** | **4** | **0.0113** | **2** | **UMUC, BOY** |
| **chr9** | **22017635** | **22036646** | **19012** | **3** | **0.0004** | **2** | **UMUC, BOY** |
| **chr9** | **22028073** | **24001994** | **1973922** | **108** | **0.0310** | **2** | **T24, BOY** |
| **chr9** | **22028073** | **22036646** | **8574** | **2** | **0.0006** | **2** | **UMUC, BOY** |
| **chr9** | **22765022** | **25779422** | **3014401** | **130** | **0.0408** | **2** | **BOY, T24** |
| **chr9** | **28481319** | **28887559** | **406241** | **39** | **0.0349** | **3** | **BOY, T24, KK47** |
| **chr9** | **29003790** | **32142056** | **3138267** | **124** | **0.0056** | **3** | **T24, BOY, KK47** |
| **chr9** | **33011920** | **33052975** | **41056** | **8** | **0.0115** | **3** | **T24, BOY, KK47** |
| **chr9** | **33011920** | **38459675** | **5447756** | **630** | **0.0063** | **2** | **BOY, T24** |
| **chr10** | **1793558** | **2721219** | **927662** | **71** | **0.0092** | **5** | **BOY, KK47, UMUC, T24, J82** |
| **chr10** | **8557285** | **11098976** | **2541692** | **106** | **0.0068** | **5** | **BOY, KK47, UMUC, T24, J82** |
| **chr10** | **19216922** | **21070677** | **1853756** | **120** | **0.0223** | **5** | **KK47, BOY, UMUC, T24, J82** |
| **chr10** | **27367081** | **28895600** | **1528520** | **143** | **0.0417** | **4** | **BOY, UMUC, T24, J82** |
|  |  |  |  |  |  |  |  |

| **Table SII (continued)** | | | | | | | |
| --- | --- | --- | --- | --- | --- | --- | --- |
| **chr10** | **36132219** | **38217157** | **2084939** | **87** | **0.0258** | **4** | **BOY, UMUC, J82, T24** |
| **chr10** | **38414338** | **38649232** | **234895** | **17** | **0.0230** | **4** | **BOY, T24, UMUC, J82** |
| **chr10** | **43881738** | **135474940** | **91593203** | **7773** | **0.0158** | **3** | **J82, BOY, T24** |
| **chr10** | **43881738** | **52433131** | **8551394** | **496** | **0.0238** | **2** | **J82, BOY** |
| **chr10** | **46975957** | **47691426** | **715470** | **35** | **0.0257** | **3** | **T24, UMUC, BOY** |
| **chr10** | **46975957** | **47148690** | **172734** | **23** | **0.0118** | **2** | **T24, UMUC** |
| **chr10** | **52825684** | **69408680** | **16582997** | **1180** | **0.0151** | **4** | **J82, BOY, T24, UMUC** |
| **chr10** | **53874480** | **54703622** | **829143** | **57** | **0.0052** | **4** | **J82, BOY, UMUC, T24** |
| **chr10** | **55329473** | **59326770** | **3997298** | **245** | **0.0125** | **2** | **J82, BOY** |
| **chr10** | **59452386** | **65872013** | **6419628** | **485** | **0.0116** | **4** | **J82, BOY, UMUC, T24** |
| **chr10** | **66314632** | **68348597** | **2033966** | **123** | **0.0167** | **2** | **J82, BOY** |
| **chr10** | **69177051** | **69408680** | **231630** | **26** | **0.0235** | **2** | **J82, BOY** |
| **chr10** | **69177051** | **69227578** | **50528** | **8** | **0.0177** | **2** | **J82, BOY** |
| **chr10** | **75780534** | **78844660** | **3064127** | **297** | **0.0459** | **3** | **J82, BOY, T24** |
| **chr10** | **83555271** | **88868693** | **5313423** | **430** | **0.0338** | **2** | **J82, BOY** |
| **chr10** | **83739042** | **84898559** | **1159518** | **121** | **0.0206** | **2** | **J82, BOY** |
| **chr10** | **99928725** | **101078949** | **1150225** | **120** | **0.0244** | **2** | **J82, BOY** |
| **chr10** | **105307650** | **134901269** | **29593620** | **2467** | **0.0055** | **3** | **T24, J82, BOY** |
| **chr10** | **106460747** | **111578843** | **5118097** | **274** | **0.0017** | **3** | **T24, J82, BOY** |
| **chr10** | **109754386** | **109905028** | **150643** | **7** | **0.0016** | **4** | **KK47, T24, J82, BOY** |
| **chr10** | **114298037** | **114745557** | **447521** | **43** | **0.0290** | **3** | **BOY, T24, J82** |
| **chr10** | **116729937** | **117705693** | **975757** | **104** | **0.0076** | **3** | **T24, J82, BOY** |
| **chr10** | **122115241** | **123083015** | **967775** | **100** | **0.0033** | **3** | **T24, J82, BOY** |
| **chr10** | **124528591** | **124900485** | **371895** | **46** | **0.0017** | **2** | **J82, T24** |
| **chr10** | **130074979** | **133646021** | **3571043** | **275** | **0.0044** | **3** | **T24, J82, BOY** |
| **chr11** | **2899703** | **13408265** | **10508563** | **1144** | **0.0248** | **2** | **UMUC, T24** |
| **chr11** | **21995188** | **25241971** | **3246784** | **179** | **0.0099** | **4** | **KK47, T24, UMUC, BOY** |
| **chr11** | **22649468** | **23234950** | **585483** | **35** | **0.0446** | **5** | **KK47, T24, UMUC, BOY, J82** |
| **chr11** | **24165461** | **24544788** | **379328** | **16** | **0.0239** | **5** | **KK47, UMUC, T24, BOY, J82** |
| **chr11** | **25622310** | **27555052** | **1932743** | **132** | **0.0229** | **2** | **T24, UMUC** |
| **chr11** | **28963825** | **29607175** | **643351** | **26** | **0.0333** | **3** | **T24, UMUC, BOY** |
| **chr11** | **51387723** | **55411099** | **4023377** | **51** | **0.0133** | **4** | **BOY, KK47, T24, J82** |
| **chr11** | **55367954** | **55411099** | **43146** | **7** | **0.0032** | **4** | **BOY, T24, J82, KK47** |
|  |  |  |  |  |  |  |  |

| **Table SII (continued)** | | | | | | | |
| --- | --- | --- | --- | --- | --- | --- | --- |
| **chr11** | **55416598** | **56433880** | **1017283** | **153** | **0.0337** | **4** | **BOY, KK47, T24, J82** |
| **chr11** | **55416598** | **55438614** | **22017** | **4** | **0.0055** | **4** | **BOY, T24, KK47, J82** |
| **chr11** | **115109069** | **115394213** | **285145** | **35** | **0.0298** | **2** | **J82, KK47** |
| **chr11** | **131477099** | **134927255** | **3450157** | **324** | **0.0087** | **3** | **J82, UMUC, KK47** |
| **chr12** | **9637123** | **9722007** | **84885** | **4** | **0.0001** | **2** | **J82, T24** |
| **chr12** | **72962203** | **74577615** | **1615413** | **71** | **0.0339** | **3** | **BOY, T24, J82** |
| **chr12** | **84259840** | **84379367** | **119528** | **6** | **0.0380** | **3** | **BOY, T24, J82** |
| **chr12** | **85037449** | **92357488** | **7320040** | **419** | **0.0460** | **3** | **BOY, T24, J82** |
| **chr13** | **19511173** | **35533776** | **16022604** | **1540** | **0.0364** | **4** | **UMUC, BOY, T24, KK47** |
| **chr13** | **34548459** | **39253980** | **4705522** | **422** | **0.0476** | **3** | **BOY, T24, KK47** |
| **chr13** | **34548459** | **35533776** | **985318** | **45** | **0.0220** | **4** | **BOY, UMUC, T24, KK47** |
| **chr13** | **69919503** | **70009062** | **89560** | **11** | **0.0080** | **2** | **T24, BOY** |
| **chr13** | **72151525** | **72617563** | **466039** | **46** | **0.0186** | **3** | **KK47, T24, BOY** |
| **chr14** | **19116954** | **20427383** | **1310430** | **39** | **0.0129** | **3** | **KK47, J82, T24** |
| **chr14** | **20198736** | **20420990** | **222255** | **30** | **0.0081** | **3** | **KK47, J82, T24** |
| **chr14** | **25479510** | **31218265** | **5738756** | **317** | **0.0206** | **3** | **KK47, T24, J82** |
| **chr14** | **37745342** | **38047147** | **301806** | **37** | **0.0119** | **3** | **KK47, T24, J82** |
| **chr14** | **47484655** | **49462787** | **1978133** | **129** | **0.0057** | **4** | **KK47, T24, J82, BOY** |
| **chr14** | **106531511** | **106559244** | **27734** | **4** | **0.0225** | **3** | **T24, UMUC, J82** |
| **chr14** | **107151892** | **107190031** | **38140** | **5** | **0.0321** | **2** | **J82, T24** |
| **chr15** | **20604368** | **21272576** | **668209** | **11** | **0.0022** | **3** | **KK47, J82, T24** |
| **chr15** | **23794210** | **51391473** | **27597264** | **2698** | **0.0031** | **5** | **UMUC, BOY, KK47, J82, T24** |
| **chr15** | **25133511** | **28277399** | **3143889** | **313** | **0.0007** | **5** | **UMUC, BOY, KK47, T24, J82** |
| **chr15** | **46318868** | **48525147** | **2206280** | **149** | **0.0011** | **5** | **BOY, UMUC, KK47, J82, T24** |
| **chr15** | **53094967** | **55140592** | **2045626** | **134** | **0.0032** | **4** | **BOY, KK47, J82, T24** |
| **chr15** | **55990134** | **69829364** | **13839231** | **1569** | **0.0084** | **2** | **BOY, KK47** |
| **chr15** | **61269268** | **62363553** | **1094286** | **97** | **0.0118** | **2** | **BOY, KK47** |
| **chr15** | **65255305** | **69829364** | **4574060** | **567** | **0.0151** | **2** | **BOY, KK47** |
| **chr15** | **70136942** | **102383614** | **32246673** | **3184** | **0.0235** | **4** | **UMUC, BOY, KK47, J82** |
| **chr15** | **72051734** | **72366735** | **315002** | **42** | **0.0282** | **4** | **KK47, UMUC, BOY, J82** |
| **chr15** | **86667584** | **88690823** | **2023240** | **119** | **0.0058** | **5** | **UMUC, BOY, KK47, J82, T24** |
| **chr15** | **94086457** | **98763464** | **4677008** | **299** | **0.0027** | **5** | **UMUC, BOY, KK47, J82, T24** |
| **chr15** | **96343001** | **96547443** | **204443** | **7** | **0.0246** | **4** | **KK47, UMUC, BOY, J82** |
|  |  |  |  |  |  |  |  |

| **Table SII (continued)** | | | | | | | |
| --- | --- | --- | --- | --- | --- | --- | --- |
| **chr16** | **4654054** | **16292381** | **11638328** | **963** | **0.0264** | **3** | **BOY, UMUC, T24** |
| **chr16** | **7541962** | **7602079** | **60118** | **7** | **0.0459** | **3** | **UMUC, BOY, T24** |
| **chr16** | **10872208** | **12663872** | **1791665** | **200** | **0.0057** | **2** | **BOY, UMUC** |
| **chr16** | **25652301** | **26949340** | **1297040** | **78** | **0.0315** | **4** | **BOY, T24, UMUC, J82** |
| **chr16** | **31720884** | **33631722** | **1910839** | **60** | **0.0224** | **4** | **BOY, UMUC, T24, KK47** |
| **chr16** | **78596047** | **78925806** | **329760** | **37** | **0.0265** | **5** | **UMUC, T24, KK47, BOY, J82** |
| **chr16** | **78650642** | **78679135** | **28494** | **4** | **0.0143** | **3** | **KK47, UMUC, T24** |
| **chr16** | **78693437** | **78814526** | **121090** | **14** | **0.0376** | **4** | **UMUC, T24, BOY, KK47** |
| **chr16** | **78718540** | **78772203** | **53664** | **7** | **0.0322** | **4** | **UMUC, BOY, T24, KK47** |
| **chr16** | **78824147** | **78970864** | **146718** | **17** | **0.0402** | **3** | **UMUC, KK47, T24** |
| **chr16** | **78888214** | **78925806** | **37593** | **4** | **0.0149** | **2** | **UMUC, KK47** |
| **chr17** | **34437275** | **34480651** | **43377** | **5** | **0.0278** | **2** | **KK47, J82** |
| **chr17** | **44159603** | **44268894** | **109292** | **13** | **0.0352** | **3** | **UMUC, J82, T24** |
| **chr18** | **4173125** | **8092292** | **3919168** | **320** | **0.0270** | **4** | **BOY, KK47, UMUC, J82** |
| **chr18** | **8584950** | **9927743** | **1342794** | **138** | **0.0360** | **3** | **BOY, KK47, UMUC** |
| **chr18** | **18811636** | **33862854** | **15051219** | **1188** | **0.0416** | **4** | **UMUC, KK47, T24, J82** |
| **chr18** | **46611300** | **46725981** | **114682** | **12** | **0.0486** | **3** | **BOY, KK47, UMUC** |
| **chr18** | **49703282** | **51116793** | **1413512** | **146** | **0.0010** | **5** | **KK47, T24, UMUC, BOY, J82** |
| **chr19** | **8585783** | **12889708** | **4303926** | **503** | **0.0089** | **3** | **UMUC, T24, J82** |
| **chr19** | **11066202** | **12889708** | **1823507** | **225** | **0.0383** | **4** | **T24, UMUC, J82, KK47** |
| **chr19** | **14706691** | **14773049** | **66359** | **8** | **0.0493** | **4** | **T24, J82, BOY, KK47** |
| **chr19** | **20035023** | **21324747** | **1289725** | **85** | **0.0031** | **5** | **J82, BOY, KK47, T24, UMUC** |
| **chr19** | **28470668** | **50472270** | **22001603** | **2222** | **0.0177** | **3** | **BOY, KK47, J82** |
| **chr19** | **52091887** | **52616837** | **524951** | **69** | **0.0399** | **2** | **BOY, KK47** |
| **chr19** | **56680305** | **59063654** | **2383350** | **299** | **0.0392** | **3** | **J82, KK47, BOY** |
| **chr20** | **1558179** | **1581099** | **22921** | **3** | **0.0001** | **3** | **KK47, J82, UMUC** |
| **chr20** | **14762783** | **14763183** | **401** | **1** | **0.0404** | **4** | **KK47, BOY, T24, J82** |
| **chr20** | **14762783** | **14838417** | **75635** | **8** | **0.0430** | **2** | **BOY, KK47** |
| **chr20** | **14762783** | **14775113** | **12331** | **2** | **0.0077** | **2** | **BOY, KK47** |
| **chr20** | **14943955** | **14972395** | **28441** | **4** | **0.0188** | **4** | **BOY, KK47, J82, T24** |
| **chr20** | **15005858** | **15088430** | **82573** | **9** | **0.0462** | **2** | **KK47, BOY** |
| **chr20** | **15005858** | **15041679** | **35822** | **5** | **0.0131** | **2** | **BOY, KK47** |
| **chr21** | **10909795** | **11176458** | **266664** | **19** | **0.0015** | **4** | **KK47, J82, BOY, T24** |
|  |  |  |  |  |  |  |  |

| **Table SII (continued)** | | | | | | | |
| --- | --- | --- | --- | --- | --- | --- | --- |
| **chr21** | **14412889** | **48090458** | **33677570** | **3334** | **0.0103** | **3** | **J82, BOY, KK47** |
| **chr21** | **15477476** | **32182162** | **16704687** | **1315** | **0.0067** | **4** | **J82, BOY, KK47, T24** |
| **chr21** | **15893261** | **19253623** | **3360363** | **286** | **0.0075** | **4** | **J82, BOY, KK47, T24** |
| **chr21** | **17677596** | **17982381** | **304786** | **56** | **0.0041** | **4** | **J82, BOY, KK47, T24** |
| **chr21** | **18668900** | **18867755** | **198856** | **22** | **0.0107** | **2** | **J82, BOY** |
| **chr21** | **19982061** | **23663369** | **3681309** | **246** | **0.0056** | **4** | **BOY, J82, KK47, T24** |
| **chr21** | **29943383** | **31235226** | **1291844** | **141** | **0.0114** | **4** | **J82, BOY, KK47, T24** |
| **chr21** | **31541941** | **32182162** | **640222** | **115** | **0.0043** | **3** | **J82, BOY, KK47** |
| **chr21** | **32635242** | **32693011** | **57770** | **10** | **0.0064** | **4** | **T24, J82, BOY, KK47** |
| **chr22** | **16124018** | **17468723** | **1344706** | **39** | **0.0310** | **4** | **KK47, J82, BOY, UMUC** |
| **chr22** | **24347759** | **24395505** | **47747** | **10** | **0.0100** | **3** | **J82, BOY, T24** |
| **chr22** | **33283949** | **35350229** | **2066281** | **158** | **0.0031** | **5** | **J82, KK47, T24, BOY, UMUC** |
| **chr22** | **36690476** | **36744794** | **54319** | **8** | **0.0429** | **3** | **J82, KK47, UMUC** |
| **chr22** | **47152681** | **49799177** | **2646497** | **246** | **0.0036** | **5** | **J82, KK47, T24, BOY, UMUC** |
| **chrX** | **6754942** | **6872470** | **117529** | **4** | **0.0380** | **2** | **KK47, T24** |
| **chrX** | **33146413** | **37042453** | **3896041** | **203** | **0.0159** | **2** | **T24, UMUC** |
| **chrX** | **33146413** | **57931902** | **24785490** | **1872** | **0.0120** | **2** | **T24, UMUC** |
| **chrX** | **39913059** | **41437948** | **1524890** | **123** | **0.0144** | **2** | **UMUC, T24** |
| **chrX** | **45298691** | **46699336** | **1400646** | **94** | **0.0092** | **2** | **T24, UMUC** |
| **chrX** | **45298691** | **49627415** | **4328725** | **441** | **0.0074** | **2** | **UMUC, T24** |
| **chrX** | **47422052** | **49134352** | **1712301** | **238** | **0.0174** | **2** | **UMUC, T24** |
| **chrX** | **47696281** | **48306301** | **610021** | **60** | **0.0182** | **2** | **T24, UMUC** |
| **chrX** | **52954320** | **54868530** | **1914211** | **191** | **0.0084** | **2** | **UMUC, T24** |
| **chrX** | **53228134** | **53562651** | **334518** | **38** | **0.0360** | **2** | **UMUC, T24** |
| **chrX** | **89049372** | **92297859** | **3248488** | **100** | **0.0437** | **2** | **T24, KK47** |
| **chrX** | **91030709** | **91046843** | **16135** | **3** | **0.0051** | **4** | **KK47, T24, BOY, UMUC** |
| **chrX** | **91598450** | **91830282** | **231833** | **14** | **0.0074** | **2** | **T24, KK47** |
| **chrX** | **104491825** | **104649726** | **157902** | **20** | **0.0021** | **2** | **T24, UMUC** |
| **chrY** | **2650250** | **2664464** | **14215** | **5** | **0.0003** | **3** | **UMUC, KK47, BOY** |
| **chrY** | **2650250** | **9901455** | **7251206** | **502** | **0.0001** | **3** | **UMUC, KK47, BOY** |
| **chrY** | **2685925** | **2737677** | **51753** | **15** | **0.0003** | **3** | **UMUC, BOY, KK47** |
| **chrY** | **2685925** | **2709494** | **23570** | **6** | **0.0003** | **3** | **UMUC, BOY, KK47** |
| **chrY** | **2685925** | **2696234** | **10310** | **3** | **0.0000** | **3** | **UMUC, BOY, KK47** |
|  |  |  |  |  |  |  |  |

| **Table SII (continued)** | | | | | | | |
| --- | --- | --- | --- | --- | --- | --- | --- |
| **chrY** | **2713429** | **2737677** | **24249** | **8** | **0.0002** | **3** | **UMUC, KK47, BOY** |
| **chrY** | **2747241** | **2757393** | **10153** | **3** | **0.0002** | **3** | **UMUC, KK47, BOY** |
| **chrY** | **2776373** | **2843672** | **67300** | **15** | **0.0002** | **3** | **UMUC, KK47, BOY** |
| **chrY** | **2867407** | **2911461** | **44055** | **8** | **0.0001** | **3** | **UMUC, KK47, BOY** |
| **chrY** | **3258261** | **6414649** | **3156389** | **92** | **0.0038** | **4** | **KK47, BOY, UMUC, T24** |
| **chrY** | **3566503** | **3703799** | **137297** | **7** | **0.0025** | **4** | **KK47, BOY, UMUC, T24** |
| **chrY** | **4634387** | **4976326** | **341940** | **17** | **0.0046** | **4** | **KK47, BOY, UMUC, T24** |
| **chrY** | **6636521** | **6735778** | **99258** | **10** | **0.0003** | **3** | **UMUC, KK47, BOY** |
| **chrY** | **6751571** | **6767086** | **15516** | **4** | **0.0141** | **3** | **UMUC, KK47, BOY** |
| **chrY** | **6777051** | **6794496** | **17446** | **6** | **0.0003** | **3** | **KK47, UMUC, BOY** |
| **chrY** | **6818107** | **6834630** | **16524** | **3** | **0.0002** | **3** | **UMUC, BOY, KK47** |
| **chrY** | **6860956** | **6896891** | **35936** | **9** | **0.0002** | **3** | **UMUC, BOY, KK47** |
| **chrY** | **6947524** | **6954103** | **6580** | **3** | **0.0093** | **3** | **UMUC, BOY, KK47** |
| **chrY** | **6985024** | **7018379** | **33356** | **3** | **0.0001** | **3** | **KK47, BOY, UMUC** |
| **chrY** | **7057276** | **7136686** | **79411** | **8** | **0.0019** | **3** | **KK47, UMUC, BOY** |
| **chrY** | **7153162** | **7165154** | **11993** | **3** | **0.0001** | **3** | **UMUC, KK47, BOY** |
| **chrY** | **7229297** | **7280982** | **51686** | **9** | **0.0002** | **3** | **UMUC, KK47, BOY** |
| **chrY** | **7382714** | **7571465** | **188752** | **9** | **0.0001** | **3** | **KK47, UMUC, BOY** |
| **chrY** | **7382714** | **7410210** | **27497** | **3** | **0.0001** | **3** | **KK47, UMUC, BOY** |
| **chrY** | **7584971** | **7613706** | **28736** | **7** | **0.0039** | **3** | **BOY, UMUC, KK47** |
| **chrY** | **7622495** | **7667134** | **44640** | **10** | **0.0002** | **3** | **BOY, UMUC, KK47** |
| **chrY** | **7622495** | **7633608** | **11114** | **3** | **0.0001** | **3** | **UMUC, KK47, BOY** |
| **chrY** | **7679871** | **7729911** | **50041** | **10** | **0.0004** | **2** | **UMUC, KK47** |
| **chrY** | **7716065** | **7729911** | **13847** | **4** | **0.0042** | **3** | **KK47, BOY, UMUC** |
| **chrY** | **7767809** | **7794150** | **26342** | **5** | **0.0001** | **3** | **UMUC, KK47, BOY** |
| **chrY** | **7810200** | **7833224** | **23025** | **3** | **0.0002** | **3** | **KK47, UMUC, BOY** |
| **chrY** | **7858699** | **7873424** | **14726** | **3** | **0.0039** | **3** | **UMUC, BOY, KK47** |
| **chrY** | **7858699** | **8118988** | **260290** | **40** | **0.0004** | **3** | **UMUC, BOY, KK47** |
| **chrY** | **7858699** | **9901455** | **2042757** | **180** | **0.0004** | **3** | **UMUC, BOY, KK47** |
| **chrY** | **7887466** | **7934113** | **46648** | **5** | **0.0001** | **3** | **UMUC, KK47, BOY** |
| **chrY** | **8170556** | **8227173** | **56618** | **11** | **0.0003** | **3** | **UMUC, KK47, BOY** |
| **chrY** | **8200690** | **8227173** | **26484** | **5** | **0.0006** | **3** | **UMUC, KK47, BOY** |
| **chrY** | **8241778** | **8251605** | **9828** | **3** | **0.0001** | **3** | **KK47, UMUC, BOY** |
|  |  |  |  |  |  |  |  |

| **Table SII (continued)** | | | | | | | |
| --- | --- | --- | --- | --- | --- | --- | --- |
| **chrY** | **8264128** | **8288204** | **24077** | **5** | **0.0004** | **3** | **UMUC, KK47, BOY** |
| **chrY** | **8297073** | **8398218** | **101146** | **7** | **0.0004** | **3** | **BOY, UMUC, KK47** |
| **chrY** | **8426304** | **8542152** | **115849** | **24** | **0.0003** | **3** | **UMUC, BOY, KK47** |
| **chrY** | **8430237** | **8453807** | **23571** | **6** | **0.0002** | **3** | **UMUC, BOY, KK47** |
| **chrY** | **8462995** | **8491784** | **28790** | **8** | **0.0000** | **3** | **UMUC, KK47, BOY** |
| **chrY** | **8500452** | **8542152** | **41701** | **7** | **0.0000** | **3** | **UMUC, KK47, BOY** |
| **chrY** | **8554017** | **8592893** | **38877** | **5** | **0.0089** | **3** | **UMUC, BOY, KK47** |
| **chrY** | **8634995** | **8670300** | **35306** | **8** | **0.0003** | **3** | **UMUC, KK47, BOY** |
| **chrY** | **8768516** | **8783485** | **14970** | **4** | **0.0001** | **3** | **UMUC, BOY, KK47** |
| **chrY** | **8809490** | **8875334** | **65845** | **5** | **0.0006** | **3** | **UMUC, BOY, KK47** |
| **chrY** | **8907047** | **9021396** | **114350** | **3** | **0.0001** | **3** | **UMUC, BOY, KK47** |
| **chrY** | **9095057** | **9117665** | **22609** | **3** | **0.0057** | **3** | **UMUC, BOY, KK47** |
| **chrY** | **9126838** | **9148477** | **21640** | **4** | **0.0000** | **3** | **UMUC, KK47, BOY** |
| **chrY** | **9386972** | **9650335** | **263364** | **10** | **0.0003** | **3** | **UMUC, BOY, KK47** |
| **chrY** | **9769312** | **9901455** | **132144** | **8** | **0.0001** | **3** | **UMUC, BOY, KK47** |
| **chrY** | **9967872** | **10035700** | **67829** | **8** | **0.0039** | **5** | **BOY, KK47, T24, UMUC, J82** |
| **chrY** | **10026079** | **10035700** | **9622** | **3** | **0.0023** | **3** | **T24, KK47, BOY** |
| **chrY** | **13902524** | **14022641** | **120118** | **6** | **0.0022** | **2** | **BOY, UMUC** |
| **chrY** | **13992104** | **14022641** | **30538** | **2** | **0.0011** | **3** | **BOY, UMUC, KK47** |
| **chrY** | **14076720** | **28571198** | **14494479** | **756** | **0.0005** | **3** | **UMUC, KK47, BOY** |
| **chrY** | **14108063** | **14128187** | **20125** | **3** | **0.0079** | **3** | **UMUC, BOY, KK47** |
| **chrY** | **14141586** | **14262355** | **120770** | **7** | **0.0040** | **3** | **KK47, UMUC, BOY** |
| **chrY** | **14491798** | **14512933** | **21136** | **3** | **0.0032** | **3** | **KK47, UMUC, BOY** |
| **chrY** | **14520994** | **14591426** | **70433** | **5** | **0.0002** | **3** | **KK47, UMUC, BOY** |
| **chrY** | **14627389** | **14918377** | **290989** | **33** | **0.0002** | **3** | **UMUC, BOY, KK47** |
| **chrY** | **14627389** | **14655842** | **28454** | **3** | **0.0000** | **3** | **UMUC, BOY, KK47** |
| **chrY** | **14777114** | **14976318** | **199205** | **36** | **0.0008** | **3** | **UMUC, BOY, KK47** |
| **chrY** | **14870203** | **14888745** | **18543** | **4** | **0.0001** | **3** | **UMUC, BOY, KK47** |
| **chrY** | **14945057** | **14971968** | **26912** | **6** | **0.0001** | **3** | **UMUC, KK47, BOY** |
| **chrY** | **14978730** | **14988536** | **9807** | **3** | **0.0004** | **3** | **UMUC, KK47, BOY** |
| **chrY** | **15017258** | **15027133** | **9876** | **3** | **0.0002** | **3** | **UMUC, KK47, BOY** |
| **chrY** | **15042259** | **15140303** | **98045** | **7** | **0.0000** | **3** | **UMUC, KK47, BOY** |
| **chrY** | **15206737** | **15314734** | **107998** | **6** | **0.0001** | **3** | **UMUC, KK47, BOY** |
|  |  |  |  |  |  |  |  |

| **Table SII (continued)** | | | | | | | |
| --- | --- | --- | --- | --- | --- | --- | --- |
| **chrY** | **15327339** | **15354463** | **27125** | **3** | **0.0006** | **3** | **UMUC, BOY, KK47** |
| **chrY** | **15357074** | **15536099** | **179026** | **36** | **0.0003** | **3** | **UMUC, KK47, BOY** |
| **chrY** | **15357074** | **15394211** | **37138** | **8** | **0.0002** | **3** | **UMUC, KK47, BOY** |
| **chrY** | **15417864** | **15441195** | **23332** | **6** | **0.0004** | **3** | **UMUC, KK47, BOY** |
| **chrY** | **15474205** | **15530775** | **56571** | **14** | **0.0001** | **3** | **UMUC, KK47, BOY** |
| **chrY** | **15554201** | **15582349** | **28149** | **7** | **0.0003** | **3** | **UMUC, KK47, BOY** |
| **chrY** | **15659932** | **15843084** | **183153** | **16** | **0.0001** | **3** | **UMUC, KK47, BOY** |
| **chrY** | **15905965** | **15948891** | **42927** | **5** | **0.0001** | **3** | **UMUC, KK47, BOY** |
| **chrY** | **15972530** | **16178520** | **205991** | **22** | **0.0009** | **3** | **UMUC, BOY, KK47** |
| **chrY** | **16046278** | **16089644** | **43367** | **5** | **0.0000** | **3** | **KK47, UMUC, BOY** |
| **chrY** | **16091571** | **16135099** | **43529** | **3** | **0.0025** | **3** | **UMUC, BOY, KK47** |
| **chrY** | **16172144** | **16366779** | **194636** | **16** | **0.0003** | **3** | **UMUC, BOY, KK47** |
| **chrY** | **16172144** | **16178520** | **6377** | **3** | **0.0003** | **3** | **UMUC, BOY, KK47** |
| **chrY** | **16403818** | **16443576** | **39759** | **4** | **0.0000** | **3** | **BOY, UMUC, KK47** |
| **chrY** | **16485110** | **16553763** | **68654** | **5** | **0.0006** | **3** | **BOY, UMUC, KK47** |
| **chrY** | **16579464** | **16633944** | **54481** | **6** | **0.0001** | **3** | **UMUC, BOY, KK47** |
| **chrY** | **16642760** | **16649457** | **6698** | **3** | **0.0002** | **3** | **UMUC, KK47, BOY** |
| **chrY** | **16659821** | **16699418** | **39598** | **7** | **0.0002** | **3** | **KK47, UMUC, BOY** |
| **chrY** | **16691045** | **16699418** | **8374** | **3** | **0.0001** | **3** | **UMUC, BOY, KK47** |
| **chrY** | **16743229** | **16781360** | **38132** | **11** | **0.0008** | **3** | **UMUC, KK47, BOY** |
| **chrY** | **16786904** | **16835577** | **48674** | **11** | **0.0001** | **3** | **UMUC, BOY, KK47** |
| **chrY** | **16866756** | **16875628** | **8873** | **3** | **0.0001** | **3** | **UMUC, KK47, BOY** |
| **chrY** | **16890027** | **16923928** | **33902** | **8** | **0.0003** | **3** | **UMUC, KK47, BOY** |
| **chrY** | **16940901** | **16953545** | **12645** | **3** | **0.0001** | **3** | **UMUC, KK47, BOY** |
| **chrY** | **17078147** | **17145809** | **67663** | **5** | **0.0057** | **3** | **UMUC, BOY, KK47** |
| **chrY** | **17197713** | **17471704** | **273992** | **15** | **0.0006** | **3** | **KK47, UMUC, BOY** |
| **chrY** | **17493225** | **17663345** | **170121** | **10** | **0.0001** | **3** | **UMUC, BOY, KK47** |
| **chrY** | **17890014** | **18214698** | **324685** | **24** | **0.0011** | **3** | **UMUC, BOY, KK47** |
| **chrY** | **17890014** | **18548230** | **658217** | **39** | **0.0003** | **3** | **UMUC, BOY, KK47** |
| **chrY** | **17890014** | **17972256** | **82243** | **6** | **0.0001** | **2** | **KK47, UMUC** |
| **chrY** | **18126400** | **18214698** | **88299** | **8** | **0.0002** | **3** | **UMUC, BOY, KK47** |
| **chrY** | **18260922** | **18402003** | **141082** | **5** | **0.0002** | **3** | **UMUC, KK47, BOY** |
| **chrY** | **18260922** | **18548230** | **287309** | **8** | **0.0001** | **3** | **UMUC, KK47, BOY** |
|  |  |  |  |  |  |  |  |

| **Table SII (continued)** | | | | | | | |
| --- | --- | --- | --- | --- | --- | --- | --- |
| **chrY** | **18711887** | **18760014** | **48128** | **3** | **0.0001** | **3** | **UMUC, BOY, KK47** |
| **chrY** | **18787017** | **18816585** | **29569** | **3** | **0.0004** | **3** | **UMUC, KK47, BOY** |
| **chrY** | **18851223** | **18973555** | **122333** | **8** | **0.0001** | **3** | **KK47, UMUC, BOY** |
| **chrY** | **19007876** | **19067146** | **59271** | **5** | **0.0200** | **3** | **UMUC, KK47, BOY** |
| **chrY** | **19020739** | **19048759** | **28021** | **3** | **0.0007** | **3** | **UMUC, KK47, BOY** |
| **chrY** | **19078580** | **19105996** | **27417** | **3** | **0.0003** | **3** | **UMUC, BOY, KK47** |
| **chrY** | **19208960** | **19269144** | **60185** | **4** | **0.0001** | **3** | **UMUC, BOY, KK47** |
| **chrY** | **19405159** | **20802743** | **1397585** | **11** | **0.0001** | **3** | **UMUC, KK47, BOY** |
| **chrY** | **19405159** | **19564481** | **159323** | **10** | **0.0001** | **3** | **UMUC, KK47, BOY** |
| **chrY** | **21053153** | **21071245** | **18093** | **3** | **0.0001** | **3** | **UMUC, KK47, BOY** |
| **chrY** | **21093504** | **21205694** | **112191** | **19** | **0.0006** | **3** | **KK47, UMUC, BOY** |
| **chrY** | **21093504** | **21140975** | **47472** | **8** | **0.0001** | **3** | **UMUC, KK47, BOY** |
| **chrY** | **21157760** | **21179429** | **21670** | **4** | **0.0001** | **3** | **UMUC, KK47, BOY** |
| **chrY** | **21225393** | **21232247** | **6855** | **3** | **0.0000** | **3** | **UMUC, KK47, BOY** |
| **chrY** | **21250613** | **21287651** | **37039** | **5** | **0.0002** | **3** | **UMUC, KK47, BOY** |
| **chrY** | **21464425** | **21492099** | **27675** | **3** | **0.0002** | **3** | **UMUC, BOY, KK47** |
| **chrY** | **21623835** | **21646475** | **22641** | **5** | **0.0002** | **3** | **UMUC, KK47, BOY** |
| **chrY** | **21657025** | **21718451** | **61427** | **8** | **0.0001** | **2** | **KK47, UMUC** |
| **chrY** | **21728154** | **21742027** | **13874** | **5** | **0.0001** | **3** | **UMUC, KK47, BOY** |
| **chrY** | **21765894** | **21838355** | **72462** | **7** | **0.0004** | **3** | **UMUC, KK47, BOY** |
| **chrY** | **21765894** | **21801867** | **35974** | **5** | **0.0002** | **3** | **UMUC, KK47, BOY** |
| **chrY** | **21864070** | **21872622** | **8553** | **3** | **0.0003** | **3** | **UMUC, KK47, BOY** |
| **chrY** | **21881789** | **21925451** | **43663** | **5** | **0.0009** | **3** | **UMUC, KK47, BOY** |
| **chrY** | **21988688** | **22126142** | **137455** | **6** | **0.0002** | **3** | **UMUC, KK47, BOY** |
| **chrY** | **22083743** | **22126142** | **42400** | **3** | **0.0000** | **3** | **UMUC, KK47, BOY** |
| **chrY** | **22178720** | **22604766** | **426047** | **9** | **0.0000** | **3** | **UMUC, BOY, KK47** |
| **chrY** | **22645017** | **22700140** | **55124** | **7** | **0.0009** | **3** | **UMUC, KK47, BOY** |
| **chrY** | **22725028** | **22741767** | **16740** | **5** | **0.0003** | **3** | **UMUC, BOY, KK47** |
| **chrY** | **22750559** | **22762243** | **11685** | **3** | **0.0002** | **3** | **UMUC, KK47, BOY** |
| **chrY** | **22898822** | **22908705** | **9884** | **3** | **0.0001** | **3** | **UMUC, BOY, KK47** |
| **chrY** | **22920121** | **23150363** | **230243** | **18** | **0.0007** | **3** | **UMUC, KK47, BOY** |
| **chrY** | **22969165** | **22992417** | **23253** | **3** | **0.0001** | **3** | **UMUC, KK47, BOY** |
| **chrY** | **23195833** | **23284095** | **88263** | **7** | **0.0002** | **3** | **UMUC, BOY, KK47** |
|  |  |  |  |  |  |  |  |

| **Table SII (continued)** | | | | | | | |
| --- | --- | --- | --- | --- | --- | --- | --- |
| **chrY** | **23331457** | **23384443** | **52987** | **4** | **0.0002** | **3** | **KK47, UMUC, BOY** |
| **chrY** | **23454519** | **23535532** | **81014** | **8** | **0.0004** | **3** | **UMUC, BOY, KK47** |
| **chrY** | **23545233** | **23550672** | **5440** | **3** | **0.0239** | **3** | **UMUC, BOY, KK47** |
| **chrY** | **23559105** | **23605225** | **46121** | **10** | **0.0001** | **3** | **UMUC, KK47, BOY** |
| **chrY** | **23622599** | **23633032** | **10434** | **3** | **0.0001** | **3** | **UMUC, KK47, BOY** |
| **chrY** | **23641326** | **23649571** | **8246** | **3** | **0.0003** | **3** | **UMUC, KK47, BOY** |
| **chrY** | **23766153** | **23962272** | **196120** | **8** | **0.0002** | **3** | **UMUC, BOY, KK47** |
| **chrY** | **23971871** | **23986608** | **14738** | **3** | **0.0007** | **3** | **KK47, UMUC, BOY** |
| **chrY** | **24095754** | **24436150** | **340397** | **10** | **0.0001** | **3** | **UMUC, BOY, KK47** |
| **chrY** | **24441392** | **24457332** | **15941** | **4** | **0.0057** | **3** | **KK47, UMUC, BOY** |
| **chrY** | **24441392** | **26276134** | **1834743** | **19** | **0.0001** | **3** | **UMUC, KK47, BOY** |
| **chrY** | **24498754** | **24514534** | **15781** | **5** | **0.0001** | **3** | **UMUC, KK47, BOY** |
| **chrY** | **24517138** | **24874501** | **357364** | **3** | **0.0007** | **3** | **BOY, UMUC, KK47** |
| **chrY** | **26344619** | **28562993** | **2218375** | **13** | **0.0085** | **3** | **UMUC, BOY, KK47** |
| **chrY** | **26344619** | **59031621** | **32687003** | **29** | **0.0018** | **3** | **UMUC, BOY, KK47** |
| **chrY** | **23331457** | **23384443** | **52987** | **4** | **0.0002** | **3** | **KK47, UMUC, BOY** |
| **chrY** | **23454519** | **23535532** | **81014** | **8** | **0.0004** | **3** | **UMUC, BOY, KK47** |
| **chrY** | **23545233** | **23550672** | **5440** | **3** | **0.0239** | **3** | **UMUC, BOY, KK47** |
| **chrY** | **23559105** | **23605225** | **46121** | **10** | **0.0001** | **3** | **UMUC, KK47, BOY** |
| **chrY** | **23622599** | **23633032** | **10434** | **3** | **0.0001** | **3** | **UMUC, KK47, BOY** |
| **chrY** | **23641326** | **23649571** | **8246** | **3** | **0.0003** | **3** | **UMUC, KK47, BOY** |
| **chrY** | **23766153** | **23962272** | **196120** | **8** | **0.0002** | **3** | **UMUC, BOY, KK47** |
| **chrY** | **23971871** | **23986608** | **14738** | **3** | **0.0007** | **3** | **KK47, UMUC, BOY** |
| **chrY** | **24095754** | **24436150** | **340397** | **10** | **0.0001** | **3** | **UMUC, BOY, KK47** |
| **chrY** | **24441392** | **24457332** | **15941** | **4** | **0.0057** | **3** | **KK47, UMUC, BOY** |
