# Supplementary material for: LY6K is a novel molecular target in bladder cancer on basis of integrate genome-wide profiling
Source: Br J Cancer. 2010 Nov 9;104(2):376–86. doi: 10.1038/sj.bjc.6605990 (PMC3031884; doi:10.1038/sj.bjc.6605990)
Supplement: Supplementary Table SIII [file 6605990x3.doc]

| **Table SIII Up-regulated genes in the LY6K transfectant** | | | | | |
| --- | --- | --- | --- | --- | --- |
| **No.** | **Symbol** | **Gene name** | **UniGene** | **Fold change** | **Function** |
| **1** | **MARCKS** | **myristoylated alanine-rich protein kinase C substrate** | **Hs.519909** | **188.2** | **Others** |
| **2** | **CX3CL1** | **chemokine (C-X3-C motif) ligand 1** | **Hs.531668** | **115.8** | **GO:0006955(immune response)** |
| **3** | **HMMR** | **hyaluronan-mediated motility receptor (RHAMM)** | **Hs.72550** | **66.2** | **Others** |
| **4** | **NCAPG** | **non-SMC condensin I complex, subunit G** | **Hs.567567** | **41.8** | **GO:0007049(cell cycle)** |
| **5** | **KIF15** | **kinesin family member 15** | **Hs.658939** | **37.0** | **GO:0007018(microtubule-based movement)** |
| **6** | **ASPM** | **asp (abnormal spindle) homolog, microcephaly associated (Drosophila)** | **Hs.121028** | **34.3** | **GO:0007049(cell cycle)** |
| **7** | **BUB1B** | **BUB1 budding uninhibited by benzimidazoles 1 homolog beta (yeast)** | **Hs.631699** | **32.7** | **GO:0008152 : metabolic process** |
| **8** | **DTL** | **denticleless homolog (Drosophila)** | **Hs.656473** | **31.6** | **GO:0006260(DNA replication)** |
| **9** | **SPC25** | **SPC25, NDC80 kinetochore complex component, homolog (S. cerevisiae)** | **Hs.421956** | **31.4** | **GO:0007049(cell cycle)** |
| **10** | **NDC80** | **NDC80 homolog, kinetochore complex component (S. cerevisiae)** | **Hs.414407** | **30.0** | **GO:0007049(cell cycle)** |
| **11** | **BIRC5** | **baculoviral IAP repeat-containing 5 (survivin)** | **Hs.514527** | **29.5** | **GO:0007049(cell cycle)** |
| **12** | **DHFR** | **dihydrofolate reductase** | **Hs.648635** | **29.4** | **GO:0008152 : metabolic process** |
| **13** | **LFNG** | **LFNG O-fucosylpeptide 3-beta-N-acetylglucosaminyltransferase** | **Hs.159142** | **29.2** | **Others** |
| **14** | **VAV3** | **vav 3 guanine nucleotide exchange factor** | **Hs.267659** | **29.0** | **GO:0007165(signal transduction)** |
| **15** | **PBK** | **PDZ binding kinase** | **Hs.104741** | **28.2** | **GO:0008152 : metabolic process** |
| **16** | **CDC45L** | **CDC45 cell division cycle 45-like (S. cerevisiae)** | **Hs.474217** | **27.5** | **GO:0007049(cell cycle)** |
| **17** | **INHBB** | **inhibin, beta B** | **Hs.1735** | **27.3** | **Others** |
| **18** | **UBE2C** | **ubiquitin-conjugating enzyme E2C** | **Hs.93002** | **26.9** | **GO:0008152 : metabolic process** |
| **19** | **DPYSL3** | **dihydropyrimidinase-like 3** | **Hs.519659** | **26.5** | **GO:0007165(signal transduction)** |
| **20** | **DEPDC1** | **DEP domain containing 1** | **Hs.445098** | **25.1** | **GO:0007165(signal transduction)** |
| **21** | **BUB1** | **BUB1 budding uninhibited by benzimidazoles 1 homolog (yeast)** | **Hs.469649** | **25.1** | **GO:0007049(cell cycle)** |
| **22** | **TOP2A** | **topoisomerase (DNA) II alpha 170kDa** | **Hs.156346** | **24.7** | **GO:0006260(DNA replication)** |
| **23** | **GINS2** | **GINS complex subunit 2 (Psf2 homolog)** | **Hs.433180** | **24.6** | **GO:0006260(DNA replication)** |
| **24** | **BLM** | **Bloom syndrome** | **Hs.169348** | **24.5** | **GO:0006260(DNA replication)** |
| **25** | **MLF1IP** | **MLF1 interacting protein** | **Hs.575032** | **24.5** | **GO:0006350(transcription)** |
| **26** | **CIT** | **citron (rho-interacting, serine/threonine kinase 21)** | **Hs.119594** | **24.4** | **GO:0007049(cell cycle)** |

| **Table SIII (continued)** | | | | | |
| --- | --- | --- | --- | --- | --- |
| **27** | **TK1** | **thymidine kinase 1, soluble** | **Hs.515122** | **24.0** | **GO:0006260(DNA replication)** |
| **28** | **DLG7** | **discs, large homolog 7 (Drosophila)** | **Hs.77695** | **23.7** | **GO:0007049(cell cycle)** |
| **29** | **RAD51AP1** | **RAD51 associated protein 1** | **Hs.591046** | **23.4** | **GO:0008152 : metabolic process** |
| **30** | **CDC2** | **cell division cycle 2, G1 to S and G2 to M** | **Hs.334562** | **22.9** | **GO:0007049(cell cycle)** |
| **31** | **TGM2** | **transglutaminase 2 (C polypeptide, protein-glutamine-gamma-glutamyltransferase)** | **Hs.517033** | **22.7** | **GO:0007165(signal transduction)** |
| **32** | **CENPF** | **centromere protein F, 350/400ka (mitosin)** | **Hs.497741** | **22.6** | **GO:0007049(cell cycle)** |
| **33** | **KIF20A** | **kinesin family member 20A** | **Hs.73625** | **21.2** | **GO:0007018(microtubule-based movement)** |
| **34** | **EDN1** | **endothelin 1** | **Hs.511899** | **21.0** | **GO:0007165(signal transduction)** |
| **35** | **ACOT11** | **acyl-CoA thioesterase 11** | **Hs.234786** | **20.6** | **GO:0007165(signal transduction)** |
| **36** | **TTK** | **TTK protein kinase** | **Hs.169840** | **20.5** | **GO:0007049(cell cycle)** |
| **37** | **NFE2L3** | **nuclear factor (erythroid-derived 2)-like 3** | **Hs.404741** | **20.1** | **GO:0006350(transcription)** |
| **38** | **RTN4RL1** | **reticulon 4 receptor-like 1** | **Hs.22917** | **19.8** | **Others** |
| **39** | **ZWINT** | **ZW10 interactor** | **Hs.591363** | **19.6** | **GO:0007049(cell cycle)** |
| **40** | **CHAF1B** | **chromatin assembly factor 1, subunit B (p60)** | **Hs.75238** | **19.4** | **GO:0006260(DNA replication)** |
| **41** | **TYMS** | **thymidylate synthetase** | **Hs.592338** | **19.2** | **GO:0006260(DNA replication)** |
| **42** | **PRIM1** | **primase, DNA, polypeptide 1 (49kDa)** | **Hs.534339** | **17.9** | **GO:0006260(DNA replication)** |
| **43** | **TUBA1B** | **tubulin, alpha 1b** | **Hs.524390** | **17.9** | **GO:0007018(microtubule-based movement)** |
| **44** | **AMIGO2** | **adhesion molecule with Ig-like domain 2** | **Hs.121520** | **17.1** | **GO:0007155(cell adhesion)** |
| **45** | **KIF2C** | **kinesin family member 2C** | **Hs.69360** | **16.7** | **GO:0007018(microtubule-based movement)** |
| **46** | **CDC25C** | **cell division cycle 25 homolog C (S. pombe)** | **Hs.656** | **16.6** | **GO:0007049(cell cycle)** |
| **47** | **SAA1** | **serum amyloid A1** | **Hs.632144** | **16.5** | **GO:0007155(cell adhesion)** |
| **48** | **CENPH** | **centromere protein H** | **Hs.631967** | **15.8** | **Others** |
| **49** | **CDC6** | **cell division cycle 6 homolog (S. cerevisiae)** | **Hs.405958** | **15.5** | **GO:0007049(cell cycle)** |
| **50** | **CCNB2** | **cyclin B2** | **Hs.194698** | **15.4** | **GO:0007049(cell cycle)** |
| **51** | **SPAG5** | **sperm associated antigen 5** | **Hs.514033** | **15.2** | **GO:0007049(cell cycle)** |
| **52** | **TPX2** | **TPX2, microtubule-associated, homolog (Xenopus laevis)** | **Hs.244580** | **14.5** | **GO:0007049(cell cycle)** |
| **53** | **NCAPH** | **non-SMC condensin I complex, subunit H** | **Hs.308045** | **14.4** | **GO:0007049(cell cycle)** |
| **54** | **INHBA** | **inhibin, beta A** | **Hs.583348** | **14.2** | **GO:0007049(cell cycle)** |

| **Table SIII (continued)** | | | | | |
| --- | --- | --- | --- | --- | --- |
| **55** | **FABP5** | **fatty acid binding protein 5 (psoriasis-associated)** | **Hs.408061** | **14.0** | **GO:0006810 : transport** |
| **56** | **CDKN3** | **cyclin-dependent kinase inhibitor 3 (CDK2-associated dual specificity phosphatase)** | **Hs.84113** | **14.0** | **GO:0007049(cell cycle)** |
| **57** | **POLQ** | **polymerase (DNA directed), theta** | **Hs.241517** | **13.4** | **GO:0006260(DNA replication)** |
| **58** | **COL4A1** | **collagen, type IV, alpha 1** | **Hs.17441** | **13.0** | **GO:0006810 : transport** |
| **59** | **NUF2** | **NUF2, NDC80 kinetochore complex component, homolog (S. cerevisiae)** | **Hs.651950** | **12.8** | **GO:0007049(cell cycle)** |
| **60** | **MAD2L1** | **MAD2 mitotic arrest deficient-like 1 (yeast)** | **Hs.591697** | **12.7** | **GO:0007049(cell cycle)** |
| **61** | **C18orf24** | **chromosome 18 open reading frame 24** | **Hs.134726** | **12.2** | **GO:0007049(cell cycle)** |
| **62** | **FOXM1** | **forkhead box M1** | **Hs.239** | **12.1** | **GO:0006350(transcription)** |
| **63** | **KLF8** | **Kruppel-like factor 8** | **Hs.646614** | **11.9** | **GO:0006350(transcription)** |
| **64** | **CDCA7** | **cell division cycle associated 7** | **Hs.470654** | **11.9** | **GO:0006350(transcription)** |
| **65** | **KCNJ16** | **potassium inwardly-rectifying channel, subfamily J, member 16** | **Hs.463985** | **11.8** | **GO:0006810 : transport** |
| **66** | **CDCA8** | **cell division cycle associated 8** | **Hs.524571** | **11.7** | **GO:0007049(cell cycle)** |
| **67** | **KIF4A** | **kinesin family member 4A** | **Hs.648326** | **11.6** | **GO:0007018(microtubule-based movement)** |
| **68** | **ZAK** | **sterile alpha motif and leucine zipper containing kinase AZK** | **Hs.444451** | **11.5** | **GO:0007049(cell cycle)** |
| **69** | **RECK** | **reversion-inducing-cysteine-rich protein with kazal motifs** | **Hs.388918** | **11.5** | **GO:0007049(cell cycle)** |
| **70** | **TROAP** | **trophinin associated protein (tastin)** | **Hs.524399** | **11.3** | **GO:0007155(cell adhesion)** |
| **71** | **GRB14** | **growth factor receptor-bound protein 14** | **Hs.411881** | **11.2** | **GO:0007165(signal transduction)** |
| **72** | **ARHGDIB** | **Rho GDP dissociation inhibitor (GDI) beta** | **Hs.504877** | **11.1** | **GO:0006955(immune response)** |
| **73** | **KIFC1** | **kinesin family member C1** | **Hs.436912** | **11.0** | **GO:0007049(cell cycle)** |
| **74** | **CCNB1** | **cyclin B1** | **Hs.23960** | **10.9** | **GO:0007049(cell cycle)** |
| **75** | **EXO1** | **exonuclease 1** | **Hs.498248** | **10.9** | **GO:0006955(immune response)** |
| **76** | **KIF23** | **kinesin family member 23** | **Hs.270845** | **10.6** | **GO:0007049(cell cycle)** |
| **77** | **FANCD2** | **Fanconi anemia, complementation group D2** | **Hs.208388** | **10.5** | **GO:0008152 : metabolic process** |
| **78** | **RAD51** | **RAD51 homolog (RecA homolog, E. coli) (S. cerevisiae)** | **Hs.631709** | **10.4** | **GO:0006260(DNA replication)** |
| **79** | **RFC3** | **replication factor C (activator 1) 3, 38kDa** | **Hs.115474** | **10.3** | **GO:0006260(DNA replication)** |
| **80** | **PTTG2** | **pituitary tumor-transforming 2** | **Hs.668806** | **10.3** | **GO:0008152 : metabolic process** |
| **81** | **CENPE** | **centromere protein E, 312kDa** | **Hs.75573** | **10.3** | **GO:0007049(cell cycle)** |
| **82** | **PYCRL** | **pyrroline-5-carboxylate reductase-like** | **Hs.165186** | **10.2** | **GO:0006810 : transport** |

| **Table SIII (continued)** | | | | | |
| --- | --- | --- | --- | --- | --- |
| **83** | **PTTG1** | **pituitary tumor-transforming 1** | **Hs.350966** | **10.0** | **GO:0006350(transcription)** |
| **84** | **ADA** | **adenosine deaminase** | **Hs.654536** | **9.9** | **GO:0006955(immune response)** |
| **85** | **ANLN** | **anillin, actin binding protein** | **Hs.62180** | **9.8** | **GO:0007049(cell cycle)** |
| **86** | **ALG6** | **asparagine-linked glycosylation 6 homolog (S. cerevisiae, alpha-1,3-glucosyltransferase)** | **Hs.657855** | **9.8** | **GO:0008152 : metabolic process** |
| **87** | **CD83** | **CD83 molecule** | **Hs.654558** | **9.8** | **GO:0006955(immune response)** |
| **88** | **KIF18A** | **kinesin family member 18A** | **Hs.301052** | **9.7** | **GO:0007018(microtubule-based movement)** |
| **89** | **LAMC2** | **laminin, gamma 2** | **Hs.591484** | **9.7** | **GO:0007155(cell adhesion)** |
| **90** | **ANXA8** | **annexin A8-like 2** | **Hs.693604** | **9.6** | **Others** |
| **91** | **E2F2** | **E2F transcription factor 2** | **Hs.194333** | **9.5** | **GO:0007049(cell cycle)** |
| **92** | **LAMB3** | **laminin, beta 3** | **Hs.497636** | **9.4** | **GO:0006810 : transport** |
| **93** | **ASRGL1** | **asparaginase like 1** | **Hs.535326** | **9.4** | **GO:0008152 : metabolic process** |
| **94** | **TNFAIP8** | **tumor necrosis factor, alpha-induced protein 8** | **Hs.656274** | **9.3** | **Others** |
| **95** | **BARD1** | **BRCA1 associated RING domain 1** | **Hs.591642** | **9.3** | **GO:0008152 : metabolic process** |
| **96** | **SNX10** | **sorting nexin 10** | **Hs.571296** | **9.2** | **GO:0006810 : transport** |
| **97** | **CENPK** | **centromere protein K** | **Hs.529778** | **9.2** | **GO:0006350(transcription)** |
| **98** | **PRC1** | **protein regulator of cytokinesis 1** | **Hs.567385** | **9.2** | **GO:0007049(cell cycle)** |
| **99** | **RIPK4** | **receptor-interacting serine-threonine kinase 4** | **Hs.517310** | **9.1** | **GO:0008152 : metabolic process** |
| **100** | **TUBA1C** | **tubulin, alpha 1c** | **Hs.652390** | **9.1** | **GO:0007018(microtubule-based movement)** |
| **101** | **PLK1** | **polo-like kinase 1 (Drosophila)** | **Hs.592049** | **8.9** | **GO:0007049(cell cycle)** |
| **102** | **AURKB** | **aurora kinase B** | **Hs.442658** | **8.8** | **GO:0007049(cell cycle)** |
| **103** | **DIDO1** | **death inducer-obliterator 1** | **Hs.517172** | **8.8** | **GO:0006350(transcription)** |
| **104** | **PTTG3** | **pituitary tumor-transforming 3** | **Hs.647156** | **8.5** | **GO:0008152 : metabolic process** |
| **105** | **HMGB2** | **high-mobility group box 2** | **Hs.434953** | **8.4** | **GO:0006260(DNA replication)** |
| **106** | **TRAIP** | **TRAF interacting protein** | **Hs.517972** | **8.3** | **GO:0007165(signal transduction)** |
| **107** | **PLCH2** | **phospholipase C, eta 2** | **Hs.170156** | **8.3** | **GO:0007165(signal transduction)** |
| **108** | **GIT2** | **G protein-coupled receptor kinase interactor 2** | **Hs.434996** | **8.2** | **GO:0007165(signal transduction)** |
| **109** | **ID4** | **inhibitor of DNA binding 4, dominant negative helix-loop-helix protein** | **Hs.519601** | **8.2** | **GO:0006350(transcription)** |
| **110** | **RFESD** | **Rieske (Fe-S) domain containing** | **Hs.399758** | **8.1** | **GO:0006810 : transport** |

| **Table SIII (continued)** | | | | | |
| --- | --- | --- | --- | --- | --- |
| **111** | **ATP8B3** | **ATPase, class I, type 8B, member 3** | **Hs.306212** | **8.1** | **GO:0006810 : transport** |
| **112** | **HMGN2** | **high-mobility group nucleosomal binding domain 2** | **Hs.181163** | **8.0** | **GO:0006350(transcription)** |
| **113** | **ZNF239** | **zinc finger protein 239** | **Hs.25040** | **8.0** | **GO:0006350(transcription)** |
| **114** | **CDCA5** | **cell division cycle associated 5** | **Hs.434886** | **8.0** | **GO:0007049(cell cycle)** |
| **115** | **LIG1** | **ligase I, DNA, ATP-dependent** | **Hs.1770** | **8.0** | **GO:0006260(DNA replication)** |
| **116** | **ACSS1** | **acyl-CoA synthetase short-chain family member 1** | **Hs.529353** | **7.9** | **GO:0008152 : metabolic process** |
| **117** | **ORC3L** | **origin recognition complex, subunit 3-like (yeast)** | **Hs.410228** | **7.9** | **GO:0006260(DNA replication)** |
| **118** | **LMCD1** | **LIM and cysteine-rich domains 1** | **Hs.475353** | **7.9** | **GO:0006350(transcription)** |
| **119** | **IDH2** | **isocitrate dehydrogenase 2 (NADP+), mitochondrial** | **Hs.596461** | **7.9** | **GO:0008152 : metabolic process** |
| **120** | **WHSC1** | **Wolf-Hirschhorn syndrome candidate 1** | **Hs.113876** | **7.8** | **GO:0006350(transcription)** |
| **121** | **GTSE1** | **G-2 and S-phase expressed 1** | **Hs.386189** | **7.8** | **GO:0007049(cell cycle)** |
| **122** | **RP5-1022P6.2** | **hypothetical protein KIAA1434** | **Hs.636359** | **7.8** | **GO:0008152 : metabolic process** |
| **123** | **KNTC1** | **kinetochore associated 1** | **Hs.300559** | **7.7** | **GO:0008152 : metabolic process** |
| **124** | **AREG** | **amphiregulin (schwannoma-derived growth factor)** | **Hs.270833** | **7.7** | **GO:0007165(signal transduction)** |
| **125** | **MTHFD1L** | **methylenetetrahydrofolate dehydrogenase (NADP+ dependent) 1-like** | **Hs.591343** | **7.6** | **GO:0008152 : metabolic process** |
| **126** | **MYBL2** | **v-myb myeloblastosis viral oncogene homolog (avian)-like 2** | **Hs.179718** | **7.6** | **GO:0007049(cell cycle)** |
| **127** | **ELAC1** | **elaC homolog 1 (E. coli)** | **Hs.657360** | **7.6** | **GO:0008152 : metabolic process** |
| **128** | **ID1** | **inhibitor of DNA binding 1, dominant negative helix-loop-helix protein** | **Hs.504609** | **7.6** | **GO:0006350(transcription)** |
| **129** | **EXPH5** | **exophilin 5** | **Hs.269591** | **7.6** | **GO:0006810 : transport** |
| **130** | **NCAPD2** | **non-SMC condensin I complex, subunit D2** | **Hs.5719** | **7.5** | **GO:0007049(cell cycle)** |
| **131** | **MCM5** | **minichromosome maintenance complex component 5** | **Hs.517582** | **7.5** | **GO:0007049(cell cycle)** |
| **132** | **OIP5** | **Opa interacting protein 5** | **Hs.661645** | **7.5** | **Others** |
| **133** | **USP18** | **ubiquitin specific peptidase 18** | **Hs.38260** | **7.5** | **GO:0008152 : metabolic process** |
| **134** | **SGOL2** | **shugoshin-like 2 (S. pombe)** | **Hs.655182** | **7.5** | **GO:0007049(cell cycle)** |
| **135** | **JAG1** | **jagged 1 (Alagille syndrome)** | **Hs.224012** | **7.4** | **GO:0007165(signal transduction)** |
| **136** | **ZNF93** | **zinc finger protein 93** | **Hs.301059** | **7.4** | **GO:0006350(transcription)** |
| **137** | **NUDT1** | **nudix (nucleoside diphosphate linked moiety X)-type motif 1** | **Hs.534331** | **7.4** | **GO:0008152 : metabolic process** |
| **138** | **IGF2** | **insulin-like growth factor 2 (somatomedin A)** | **Hs.523414** | **7.4** | **GO:0007049(cell cycle)** |
| **139** | **FLJ10986** | **FGGY carbohydrate kinase domain containing** | **Hs.444301** | **7.4** | **GO:0008152 : metabolic process** |

| **Table SIII (continued)** | | | | | |
| --- | --- | --- | --- | --- | --- |
| **140** | **ENST00000270201** |  | **Hs.473611** | **7.3** | **Others** |
| **141** | **KIF11** | **kinesin family member 11** | **Hs.8878** | **7.3** | **GO:0007018(microtubule-based movement)** |
| **142** | **DHRS2** | **dehydrogenase/reductase (SDR family) member 2** | **Hs.272499** | **7.2** | **GO:0006810 : transport** |
| **143** | **APOBEC3G** | **apolipoprotein B mRNA editing enzyme, catalytic polypeptide-like 3G** | **Hs.660143** | **7.2** | **GO:0006350(transcription)** |
| **144** | **CDCA3** | **cell division cycle associated 3** | **Hs.524216** | **7.2** | **Others** |
| **145** | **THBS1** | **thrombospondin 1** | **Hs.164226** | **7.2** | **GO:0007155(cell adhesion)** |
| **146** | **ABHD11** | **abhydrolase domain containing 11** | **Hs.647045** | **7.1** | **GO:0008152 : metabolic process** |
| **147** | **SOX9** | **SRY (sex determining region Y)-box 9 (campomelic dysplasia, autosomal sex-reversal)** | **Hs.647409** | **7.1** | **GO:0006350(transcription)** |
| **148** | **ORC6L** | **origin recognition complex, subunit 6 like (yeast)** | **Hs.49760** | **7.1** | **GO:0006260(DNA replication)** |
| **149** | **RGS2** | **regulator of G-protein signaling 2, 24kDa** | **Hs.78944** | **7.1** | **GO:0007049(cell cycle)** |
| **150** | **ENST00000368778** | **uroporphyrinogen III synthase (congenital erythropoietic porphyria)** | **Hs.501376** | **7.1** | **GO:0008152 : metabolic process** |
| **151** | **BRCA1** | **breast cancer 1, early onset** | **Hs.194143** | **7.1** | **GO:0007049(cell cycle)** |
| **152** | **SH3TC1** | **SH3 domain and tetratricopeptide repeats 1** | **Hs.479116** | **7.0** | **Others** |
| **153** | **NRG1** | **neuregulin 1** | **Hs.453951** | **7.0** | **GO:0007165(signal transduction)** |
| **154** | **CTSL2** | **cathepsin L2** | **Hs.660866** | **6.9** | **GO:0008152 : metabolic process** |
| **155** | **TUBA1A** | **tubulin, alpha 1a** | **Hs.654422** | **6.9** | **GO:0007018(microtubule-based movement)** |
| **156** | **TMPO** | **thymopoietin** | **Hs.11355** | **6.9** | **GO:0006350(transcription)** |
| **157** | **STOML1** | **stomatin (EPB72)-like 1** | **Hs.194816** | **6.8** | **Others** |
| **158** | **COL4A2** | **collagen, type IV, alpha 2** | **Hs.508716** | **6.8** | **GO:0006810 : transport** |
| **159** | **TUBA4A** | **tubulin, alpha 4a** | **Hs.75318** | **6.8** | **GO:0007018(microtubule-based movement)** |
| **160** | **TNFRSF6B** | **tumor necrosis factor receptor superfamily, member 6b, decoy** | **Hs.434878** | **6.8** | **GO:0008152 : metabolic process** |
| **161** | **EDG7** | **lysophosphatidic acid receptor 3** | **Hs.674915** | **6.7** | **GO:0007165(signal transduction)** |
| **162** | **TUBA3D** | **tubulin, alpha 3d** | **Hs.503749** | **6.7** | **GO:0007018(microtubule-based movement)** |
| **163** | **PSMB9** | **proteasome (prosome, macropain) subunit, beta type, 9 (large multifunctional peptidase 2)** | **Hs.654585** | **6.7** | **GO:0006955(immune response)** |
| **164** | **GLRX** | **glutaredoxin (thioltransferase)** | **Hs.28988** | **6.7** | **GO:0006810 : transport** |
| **165** | **RRM1** | **ribonucleotide reductase M1** | **Hs.558393** | **6.7** | **GO:0006260(DNA replication)** |
| **166** | **SFXN2** | **sideroflexin 2** | **Hs.44070** | **6.6** | **GO:0006810 : transport** |
| **167** | **PDSS1** | **prenyl (decaprenyl) diphosphate synthase, subunit 1** | **Hs.558468** | **6.6** | **GO:0008152 : metabolic process** |

| **Table SIII (continued)** | | | | | |
| --- | --- | --- | --- | --- | --- |
| **168** | **CNTNAP3** | **contactin associated protein-like 3** | **Hs.658328** | **6.6** | **GO:0007165(signal transduction)** |
| **169** | **NSBP1** | **nucleosomal binding protein 1** | **Hs.282204** | **6.6** | **GO:0006350(transcription)** |
| **170** | **CDC20** | **cell division cycle 20 homolog (S. cerevisiae)** | **Hs.524947** | **6.5** | **GO:0007049(cell cycle)** |
| **171** | **PTPRK** | **protein tyrosine phosphatase, receptor type, K** | **Hs.155919** | **6.5** | **GO:0008152 : metabolic process** |
| **172** | **E2F1** | **E2F transcription factor 1** | **Hs.654393** | **6.5** | **GO:0007049(cell cycle)** |
| **173** | **XRCC6BP1** | **XRCC6 binding protein 1** | **Hs.61188** | **6.5** | **GO:0008152 : metabolic process** |
| **174** | **PAPSS2** | **3'-phosphoadenosine 5'-phosphosulfate synthase 2** | **Hs.524491** | **6.5** | **GO:0008152 : metabolic process** |
| **175** | **TFPI2** | **tissue factor pathway inhibitor 2** | **Hs.438231** | **6.4** | **Others** |
| **176** | **BMF** | **Bcl2 modifying factor** | **Hs.591104** | **6.4** | **Others** |
| **177** | **LDHD** | **lactate dehydrogenase D** | **Hs.380929** | **6.4** | **GO:0006810 : transport** |
| **178** | **PPP2R2C** | **protein phosphatase 2 (formerly 2A), regulatory subunit B, gamma isoform** | **Hs.479069** | **6.3** | **GO:0007165(signal transduction)** |
| **179** | **CD24** | **CD24 molecule** | **Hs.644105** | **6.3** | **GO:0006955(immune response)** |
| **180** | **MCM6** | **minichromosome maintenance complex component 6** | **Hs.444118** | **6.3** | **GO:0006260(DNA replication)** |
| **181** | **DHODH** | **dihydroorotate dehydrogenase** | **Hs.654427** | **6.3** | **GO:0008152 : metabolic process** |
| **182** | **ZNF331** | **zinc finger protein 331** | **Hs.185674** | **6.3** | **GO:0006350(transcription)** |
| **183** | **MMD** | **monocyte to macrophage differentiation-associated** | **Hs.463483** | **6.2** | **Others** |
| **184** | **SOX4** | **SRY (sex determining region Y)-box 4** | **Hs.643910** | **6.2** | **GO:0006350(transcription)** |
| **185** | **MAP3K5** | **mitogen-activated protein kinase kinase kinase 5** | **Hs.186486** | **6.2** | **GO:0007165(signal transduction)** |
| **186** | **CRADD** | **CASP2 and RIPK1 domain containing adaptor with death domain** | **Hs.591016** | **6.2** | **GO:0007165(signal transduction)** |
| **187** | **NLN** | **neurolysin (metallopeptidase M3 family)** | **Hs.247460** | **6.2** | **GO:0008152 : metabolic process** |
| **188** | **TRMT5** | **TRM5 tRNA methyltransferase 5 homolog (S. cerevisiae)** | **Hs.380159** | **6.1** | **GO:0008152 : metabolic process** |
| **189** | **RTN4R** | **reticulon 4 receptor** | **Hs.30868** | **6.1** | **Others** |
| **190** | **E2F7** | **E2F transcription factor 7** | **Hs.416375** | **6.1** | **GO:0007049(cell cycle)** |
| **191** | **COQ3** | **coenzyme Q3 homolog, methyltransferase (S. cerevisiae)** | **Hs.653253** | **6.1** | **GO:0008152 : metabolic process** |
| **192** | **POLR3G** | **polymerase (RNA) III (DNA directed) polypeptide G (32kD)** | **Hs.282387** | **6.1** | **GO:0006350(transcription)** |
| **193** | **ITGA6** | **integrin, alpha 6** | **Hs.133397** | **6.1** | **GO:0007165(signal transduction)** |
| **194** | **EBI3** | **Epstein-Barr virus induced gene 3** | **Hs.501452** | **6.1** | **GO:0006955(immune response)** |
| **195** | **COL6A1** | **collagen, type VI, alpha 1** | **Hs.474053** | **6.1** | **GO:0006810 : transport** |
| **196** | **ENST00000329156** | **similar to high-mobility group box 3** | **Hs.659876** | **6.1** | **GO:0006350(transcription)** |

| **Table SIII (continued)** | | | | | |
| --- | --- | --- | --- | --- | --- |
| **197** | **PRSS16** | **protease, serine, 16 (thymus)** | **Hs.274407** | **6.1** | **GO:0008152 : metabolic process** |
| **198** | **RFC2** | **replication factor C (activator 1) 2, 40kDa** | **Hs.647062** | **6.1** | **GO:0006260(DNA replication)** |
| **199** | **WDHD1** | **WD repeat and HMG-box DNA binding protein 1** | **Hs.385998** | **6.0** | **GO:0006350(transcription)** |
| **200** | **MYCBP** | **c-myc binding protein** | **Hs.591506** | **6.0** | **GO:0006350(transcription)** |
| **201** | **PRSS23** | **protease, serine, 23** | **Hs.25338** | **6.0** | **GO:0008152 : metabolic process** |
| **202** | **SERPINF1** | **serpin peptidase inhibitor, clade F (alpha-2 antiplasmin, pigment epithelium derived factor), member 1** | **Hs.532768** | **6.0** | **Others** |
| **203** | **RLN2** | **relaxin 2** | **Hs.127032** | **5.9** | **Others** |
| **204** | **SGEF** | **Src homology 3 domain-containing guanine nucleotide exchange factor** | **Hs.570682** | **5.9** | **GO:0007165(signal transduction)** |
| **205** | **PKMYT1** | **protein kinase, membrane associated tyrosine/threonine 1** | **Hs.77783** | **5.9** | **GO:0007049(cell cycle)** |
| **206** | **LRP8** | **low density lipoprotein receptor-related protein 8, apolipoprotein e receptor** | **Hs.576154** | **5.9** | **GO:0007165(signal transduction)** |
| **207** | **SRBD1** | **S1 RNA binding domain 1** | **Hs.14229** | **5.8** | **GO:0008152 : metabolic process** |
| **208** | **ARHGAP11A** | **Rho GTPase activating protein 11A** | **Hs.591130** | **5.8** | **GO:0007165(signal transduction)** |
| **209** | **GSG2** | **germ cell associated 2 (haspin)** | **Hs.534059** | **5.8** | **GO:0007049(cell cycle)** |
| **210** | **FANCF** | **Fanconi anemia, complementation group F** | **Hs.632151** | **5.8** | **GO:0008152 : metabolic process** |
| **211** | **SLC2A4RG** | **SLC2A4 regulator** | **Hs.435126** | **5.8** | **GO:0006350(transcription)** |
| **212** | **MALL** | **mal, T-cell differentiation protein-like** | **Hs.185055** | **5.8** | **Others** |
| **213** | **SGOL1** | **shugoshin-like 1 (S. pombe)** | **Hs.105153** | **5.8** | **GO:0007049(cell cycle)** |
| **214** | **SPA17** | **sperm autoantigenic protein 17** | **Hs.286233** | **5.8** | **GO:0007165(signal transduction)** |
| **215** | **RIMS3** | **regulating synaptic membrane exocytosis 3** | **Hs.654808** | **5.8** | **GO:0006810 : transport** |
| **216** | **COL12A1** | **collagen, type XII, alpha 1** | **Hs.101302** | **5.7** | **GO:0007155(cell adhesion)** |
| **217** | **PBX4** | **pre-B-cell leukemia homeobox 4** | **Hs.466257** | **5.7** | **GO:0006350(transcription)** |
| **218** | **RTEL1** | **regulator of telomere elongation helicase 1** | **Hs.434878** | **5.7** | **GO:0008152 : metabolic process** |
| **219** | **ZNF25** | **zinc finger protein 25** | **Hs.499429** | **5.7** | **GO:0006350(transcription)** |
| **220** | **KREMEN1** | **kringle containing transmembrane protein 1** | **Hs.229335** | **5.7** | **GO:0007165(signal transduction)** |
| **221** | **CSRP2** | **cysteine and glycine-rich protein 2** | **Hs.530904** | **5.6** | **Others** |
| **222** | **RNASEH2A** | **ribonuclease H2, subunit A** | **Hs.532851** | **5.6** | **GO:0006260(DNA replication)** |
| **223** | **IGFBP7** | **insulin-like growth factor binding protein 7** | **Hs.479808** | **5.6** | **GO:0007049(cell cycle)** |
| **224** | **CCNF** | **cyclin F** | **Hs.1973** | **5.6** | **GO:0007049(cell cycle)** |

| **Table SIII (continued)** | | | | | |
| --- | --- | --- | --- | --- | --- |
| **225** | **TUBB2A** | **tubulin, beta 2A** | **Hs.654543** | **5.6** | **GO:0007018(microtubule-based movement)** |
| **226** | **FAS** | **Fas (TNF receptor superfamily, member 6)** | **Hs.244139** | **5.6** | **GO:0006955(immune response)** |
| **227** | **MELK** | **maternal embryonic leucine zipper kinase** | **Hs.184339** | **5.5** | **GO:0008152 : metabolic process** |
| **228** | **ZNF587** | **zinc finger protein 587** | **Hs.642598** | **5.5** | **GO:0006350(transcription)** |
| **229** | **UBE2T** | **ubiquitin-conjugating enzyme E2T (putative)** | **Hs.5199** | **5.5** | **GO:0008152 : metabolic process** |
| **230** | **PADI2** | **peptidyl arginine deiminase, type II** | **Hs.33455** | **5.5** | **GO:0008152 : metabolic process** |
| **231** | **UHRF1** | **ubiquitin-like, containing PHD and RING finger domains, 1** | **Hs.108106** | **5.4** | **GO:0006350(transcription)** |
| **232** | **HMG4L** | **high-mobility group (nonhistone chromosomal) protein 4-like** | **Hs.558624** | **5.4** | **GO:0006350(transcription)** |
| **233** | **NUB1** | **negative regulator of ubiquitin-like proteins 1** | **Hs.647082** | **5.4** | **Others** |
| **234** | **TCOF1** | **Treacher Collins-Franceschetti syndrome 1** | **Hs.519672** | **5.4** | **GO:0006810 : transport** |
| **235** | **PCGF5** | **polycomb group ring finger 5** | **Hs.500512** | **5.4** | **GO:0006350(transcription)** |
| **236** | **KIRREL2** | **kin of IRRE like 2 (Drosophila)** | **Hs.145729** | **5.4** | **GO:0007155(cell adhesion)** |
| **237** | **PDK3** | **pyruvate dehydrogenase kinase, isozyme 3** | **Hs.658190** | **5.4** | **GO:0008152 : metabolic process** |
| **238** | **LMO7** | **LIM domain 7** | **Hs.207631** | **5.3** | **GO:0008152 : metabolic process** |
| **239** | **PXDN** | **peroxidasin homolog (Drosophila)** | **Hs.332197** | **5.3** | **GO:0006955(immune response)** |
| **240** | **ALDH1A3** | **aldehyde dehydrogenase 1 family, member A3** | **Hs.459538** | **5.3** | **GO:0008152 : metabolic process** |
| **241** | **EMG1** | **EMG1 nucleolar protein homolog (S. cerevisiae)** | **Hs.558447** | **5.3** | **GO:0008152 : metabolic process** |
| **242** | **LIN9** | **lin-9 homolog (C. elegans)** | **Hs.120817** | **5.3** | **GO:0006260(DNA replication)** |
| **243** | **KCNN4** | **potassium intermediate/small conductance calcium-activated channel, subfamily N, member 4** | **Hs.10082** | **5.3** | **GO:0006810 : transport** |
| **244** | **SIN3B** | **SIN3 homolog B, transcription regulator (yeast)** | **Hs.13999** | **5.2** | **GO:0006350(transcription)** |
| **245** | **TNS3** | **tensin 3** | **Hs.520814** | **5.2** | **GO:0007165(signal transduction)** |
| **246** | **PPIH** | **peptidylprolyl isomerase H (cyclophilin H)** | **Hs.256639** | **5.2** | **GO:0008152 : metabolic process** |
| **247** | **FGF1** | **fibroblast growth factor 1 (acidic)** | **Hs.483635** | **5.2** | **GO:0007049(cell cycle)** |
| **248** | **PCNA** | **proliferating cell nuclear antigen** | **Hs.147433** | **5.2** | **GO:0007049(cell cycle)** |
| **249** | **NEBL** | **nebulette** | **Hs.5025** | **5.2** | **GO:0006810 : transport** |
| **250** | **NSL1** | **NSL1, MIND kinetochore complex component, homolog (S. cerevisiae)** | **Hs.497692** | **5.1** | **GO:0007049(cell cycle)** |
| **251** | **PAG1** | **phosphoprotein associated with glycosphingolipid microdomains 1** | **Hs.266175** | **5.1** | **GO:0006955(immune response)** |
| **252** | **ARFRP1** | **ADP-ribosylation factor related protein 1** | **Hs.389277** | **5.1** | **GO:0007165(signal transduction)** |

| **Table SIII (continued)** | | | | | |
| --- | --- | --- | --- | --- | --- |
| **253** | **C10orf125** | **chromosome 10 open reading frame 125** | **Hs.155823** | **5.1** | **GO:0006810 : transport** |
| **254** | **DERA** | **2-deoxyribose-5-phosphate aldolase homolog (C. elegans)** | **Hs.39429** | **5.1** | **GO:0008152 : metabolic process** |
| **255** | **PWP2** | **PWP2 periodic tryptophan protein homolog (yeast)** | **Hs.449076** | **5.1** | **GO:0007165(signal transduction)** |
| **256** | **C14orf130** | **chromosome 14 open reading frame 130** | **Hs.648806** | **5.1** | **GO:0008152 : metabolic process** |
| **257** | **GCA** | **grancalcin, EF-hand calcium binding protein** | **Hs.377894** | **5.1** | **Others** |
| **258** | **MGST1** | **microsomal glutathione S-transferase 1** | **Hs.389700** | **5.1** | **GO:0008152 : metabolic process** |
| **259** | **AOX1** | **aldehyde oxidase 1** | **Hs.406238** | **5.1** | **GO:0006810 : transport** |
| **260** | **C21orf6** | **RWD domain containing 2B** | **Hs.34136** | **5.1** | **Others** |
| **261** | **TACC3** | **transforming, acidic coiled-coil containing protein 3** | **Hs.104019** | **5.1** | **GO:0007049(cell cycle)** |
| **262** | **ADAM19** | **ADAM metallopeptidase domain 19 (meltrin beta)** | **Hs.483944** | **5.1** | **GO:0008152 : metabolic process** |
| **263** | **TUBB3** | **tubulin, beta 3** | **Hs.511743** | **5.1** | **GO:0007018(microtubule-based movement)** |
| **264** | **OXCT1** | **3-oxoacid CoA transferase 1** | **Hs.278277** | **5.1** | **GO:0008152 : metabolic process** |
| **265** | **MICAL2** | **microtubule associated monoxygenase, calponin and LIM domain containing 2** | **Hs.501928** | **5.0** | **GO:0006810 : transport** |
| **266** | **RPP40** | **ribonuclease P/MRP 40kDa subunit** | **Hs.511756** | **5.0** | **GO:0008152 : metabolic process** |
| **267** | **KLHL12** | **kelch-like 12 (Drosophila)** | **Hs.282878** | **5.0** | **GO:0007165(signal transduction)** |
| **268** | **LOC441795** | **similar to high-mobility group box 3** |  | **5.0** | **GO:0006350(transcription)** |
| **269** | **FBXO16** | **F-box protein 16** | **Hs.659190** | **5.0** | **GO:0008152 : metabolic process** |
